# Impact of Sustained Fructose Consumption on Gastrointestinal Function and Health in *Wistar* Rats: Glycoxidative Stress, Impaired Protein Digestion, and Shifted Fecal Microbiota

**DOI:** 10.1021/acs.jafc.3c04515

**Published:** 2023-10-20

**Authors:** Guadalupe Sánchez-Terrón, Remigio Martínez, Jorge Ruiz, Carolina Luna, Mario Estévez

**Affiliations:** † TECAL Research Group, Meat and Meat Products Research Institute (IPROCAR), Universidad de Extremadura (UEX), Cáceres 10003, Spain; ‡ Animal Health Department, Universidad of Extremadura (UEX), Cáceres 10003, Spain; § Animal Health Department, GISAZ Research Group, ENZOEM Competitive Research Unit, Universidad of Córdoba (UCO), Córdoba 14014, Spain; ∥ Emergency Unit, Servicio Extremeño de Salud, SES, Junta de Extremadura, Cáceres 10003, Spain

**Keywords:** fructose, metabolomic, microbiota, protein digestibility, protein glycoxidation

## Abstract

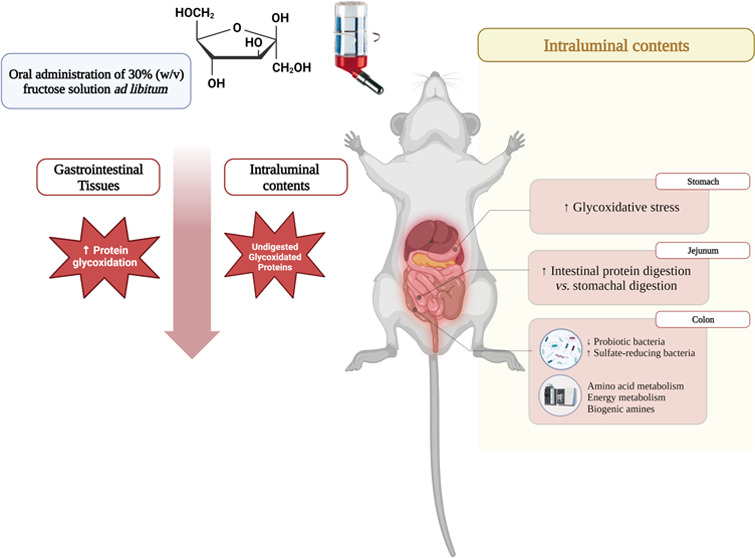

The gastrointestinal tract (GIT) is the target of assorted pathological conditions, and dietary components are known to affect its functionality and health. In previous *in vitro* studies, we observed that reducing sugars induced protein glycoxidation and impaired protein digestibility. To gain further insights into the pathophysiological effects of dietary sugars, *Wistar* rats were provided with a 30% (w/v) fructose water solution for 10 weeks. Upon slaughter, *in vivo* protein digestibility was assessed, and the entire GIT (digests and tissues) was analyzed for markers of oxidative stress and untargeted metabolomics. Additionally, the impact of sustained fructose intake on colonic microbiota was also evaluated. High fructose intake for 10 weeks decreased protein digestibility and promoted changes in the physiological digestion of proteins, enhancing intestinal digestion rather than stomach digestion. Moreover, at colonic stages, the oxidative stress was harmfully increased, and both the microbiota and the intraluminal colonic metabolome were modified.

## 1 Introduction

The World Health Organization (WHO) has recommended for more than a decade limiting free sugar intake to less than 10% of the total energy intake based on the evidence showing that a higher consumption of sugars increases the risk of metabolic diseases.^1^

Sucrose (50% fructose) is the most used sugar in the food industry. According to WHO recommendations, a healthy individual should not consume more than 25 g of sucrose per day, which corresponds to a recommended daily intake of less than 12 g.^1^ However, consumption of elevated levels of dietary fructose is currently an established daily habit through the consumption of sugar-sweetened beverages, snacks, and baked goods formulated with sucrose or commercial high-fructose corn syrup (HFCS) (55% fructose).^2^ There is a body of evidence that excessive fructose consumption is responsible for several metabolic impairments, which are associated with metabolic syndrome (MetS) due to the disturbance of liver metabolism. The main manifestations of these impairments are adiposity, dyslipidaemia, nonalcoholic fatty liver disease (NAFLD), insulin resistance, and type 2 diabetes (T2D).^3^

Besides the high caloric value of sugars (proadiposity) and their ability to induce insulin resistance (prodiabetic), the molecular basis of the noxious effects of increased levels of circulating sugar is related to the onset of oxidative stress mechanisms.^4,5^ In particular, the reactive carbonyl moiety in reducing sugars such as fructose plays a pivotal role in the pathophysiological effects of these species. Reducing sugars and reactive carbonyl species (RCS) formed from their degradation (i.e., dicarbonyls such as glyoxal and methylglyoxal) are known to induce oxidative damage to proteins and other biomolecules (glycoxidation).^6^ Protein carbonylation is an early manifestation of glycoxidation, which is known to take place, for instance, in individuals suffering from insulin resistance and enduring hyperglycemia.^7,8^ In a recent study, we were able to reproduce the entire carbonylation pathway (lysine–allysine–aminoadipic acid) in human plasma proteins under simulated hyperglycemic conditions.^9^ Protein carbonyls and other sugar-derived reactive species are implicated in the formation of advanced glycation end products (AGEs), which are accumulated in target tissues leading to physiological impairments.^10^ The ability of sugars and their dicarbonyls to induce oxidative stress in several organs such as the intestine,^11^ liver,^12^ pancreas,^13^ and brain^14^ is thought to be associated with the onset of various of the aforementioned related diseases (NALD, T2D, aging, etc.).^3^

While the impact of dietary fructose on the physiology of the liver, pancreas, and various other internal organs is well known,^3^ fructose may interact, prior to intestinal uptake and organic distribution, with other dietary components, with microbiota and epithelial cells from the GIT leading to noxious effects at this location. However, the postprandial effects of fructose consumption are poorly understood. A previous *in vitro* study revealed the severe deleterious effects of glucose on the oxidative stability and digestibility of dietary proteins when allowed to react in the pro-oxidative environment of the stomach.^15^ Under simulated physiological conditions, glucose enhances the glycoxidative damage to meat proteins, leading to impaired digestibility and a loss of nutritional value. *In vivo* studies on the impact of glucose, fructose, and other sugars with highly reactive carbonyls on the onset of luminal or tissue oxidative stress in the GIT are scarce. In a recent study, it was observed that fructose consumption led to disturbance of intestinal microbiota, and that, in turn, with abnormal immune response.^16^ The onset of enduring oxidative stress in the lumen, which may eventually transfer to the epithelium of the GIT, along with severe microbiota disturbance (dysbiosis), has been hypothesized to contribute to the onset of numerous pathological conditions, such as inflammatory bowel disease (IBD) and colorectal cancer (CRC).^17^

Given the many complex mechanisms by which fructose may affect gut health and, in turn, organic homeostasis, this study was designed to gain a deeper understanding of the effects of fructose intake on protein digestibility and the occurrence of oxidative stress using an *in vivo* model (*Wistar* rats). The impact of sustained fructose consumption on gut microbiota and colonic metabolome was also studied.

## 2 Materials and Methods

### 2.1 Chemicals

All reagents, chemicals, and standard compounds were obtained from Sigma Chemicals (Sigma-Aldrich, Stheinheim, Germany), Fisher (Fisher Scientific S.L., Madrid, Spain), and Panreac (Panreac Qumica, S.A., Barcelona, Spain). Ultrapure water was prepared using a Milli-Q water purification system (Millipore Corp., Bedford, MA).

### 2.2 Animals, Feeds, and Other Materials

*Wistar* breed rats of the *Rattus novergicus* species were used in our experiment according to Spanish legal requirements (RD 53/2013), the bioethics committee of the University of Extremadura (137-2020), and approval of the Board of Extremadura (EXP20200904). The design and performance of the experiment, including animal manipulation and euthanasia, were carried out by licensed veterinarians with all requirements by legal authority (Dirección General de Sanidad Animal de Junta de Extremadura). Twelve male rats were used in the present study. The rats were supplied and maintained during the whole assay at the Animal Facilities Service of the University of Extremadura (Cáceres, Spain), and at the beginning of the assay, they were 6–7 weeks old and weighed 186 g on average. During the entire study period, the same rodent basal feed used was the “Teklad Global Diet 2014”, supplied by ENVIGO (Madison, WI), with a crude protein content of 14.3%.

### 2.3 Experimental Design

The animals were subjected to a 1 week adaptation period. During this period, the rats were maintained in ventilated cages, with water and feed *ad libitum*, under controlled climatic conditions (20–22 °C temperature, 40–50% humidity and 12–12 h light/dark cycle). Individual identification of animals was performed during the adaptation period by means of a perforation code in the auditory pavilion.

After the adaptation period was concluded, we divided the animals into two experimental groups (*n* = 6 in each group): (i) a control group (C) that received the basal feed and drinking water during the entire assay and (ii) a fructose group (F), which consumed basal feed and 30% w/v fructose water solution. The rats coexisted in subgroups of three animals per cage. On average, rats from the F group had 9 g of fructose/kg of live bodyweight/day. The 30% (w/v) fructose solution is selected based on the literature that reported significant oxidative stress in *Wistar* rats induced by the dietary intake of such an amount of sugar.^18^ Additionally, the 30% of fructose we applied is in the range between 20% (equivalent to the top 5% of American consumers) and 63% of free fructose concentrations in the diet.^19^

The experiment was conducted for 10 weeks. The animals were visited and checked daily to ensure their safety and well-being. During the assay, food and water consumption were gravimetrically monitored every time they were filled, depending on the demand of the animals (every 2 or 3 days, approximately), and bodyweights were registered weekly (Table S2).

### 2.4 Slaughter, Necropsy and Sampling

Both food and drink were *ad libitum* available to experimental animals until slaughter. *Wistar* rats were euthanized at the end of the experimental period at an approximate age of 16–17 weeks old and an average weight of 437 g. Euthanasia was performed by exsanguination via cardiac puncture. Previously, the animals were anesthetized using 5% inhaled isoflurane. The GIT of the animals was readily dissected from corpses and clamped to avoid loss of intraluminal material. The stomach, small intestine (jejunum), cecum, and large intestine (distal colon) were aseptically sampled. Under the same conditions, the intraluminal material (digests) at each of the aforementioned locations was gently removed, dispensed in Eppendorf tubes, and stored immediately at −80 °C until analyses were performed. Feces from the rectum were also aseptically collected and stored at −80 °C until analyses were performed. Once emptied, the tissue from each location was thoroughly cleaned with cold distilled water. A portion of each location was dispensed in *Eppendorf* tubes and stored at −80 °C until analyses were performed.

### 2.5 Analytical Procedures

#### 2.5.1 Assessment of Glycoxidative Stress in Digests and Gut Tissues

##### 2.5.1.1 Protein Carbonylation

The accretion of protein carbonyls in the feeds, luminal contents, and tissues was assessed as previously described,^20^ with slight modifications. The quantification of specific protein carbonyls, namely, α-aminoadipic and γ-glutamic semialdehydes (α-AS and γ-GS, respectively), was carried out using an HPLC analysis attached to a fluoresce detector. GIT digests and tissues were thoroughly homogenized. For contents, 250 mg of the stomach, jejunum, cecum, and colon digests, as well as feces, were individually mixed and homogenized with 1 mL of PBS in Eppendorf tubes in a mixer mill. On the other hand, 500 mg of the respective tissues were homogenized with 0.5 mL of PBS. Results from the quantification of α-AS and γ-GS were expressed as total primary protein carbonyls (PPCs) as nmol carbonyl/mg protein. The remaining steps of the procedure were exactly as those reported by the above-mentioned authors.^20^

##### 2.5.1.2 Advanced Protein Oxidation Products (APOPs)

APOPs were analyzed using fluorescent spectroscopy (PerkinElmer, Beaconsfield, U.K.), as reported.^9^ Thoroughly homogenized samples were diluted with 100 mM sodium phosphate buffer, pH 7.4, with 2 M guanidine chlorhydrate. APOPs were excited at 350 nm, and the emitted fluorescence was recorded from 400 to 500 nm. The excitation and emission slits were both set to 10 nm, and the scanning speed was 500 nm/min. The fluorescence results were applied to a correction factor (Cf = Pt/Pp) where Pt is the total average of the amount of protein from all samples and Pp is the content of protein in each sample. Results are expressed as arbitrary fluorescence intensity (area units) (FU).

##### 2.5.1.3 Thiobarbituric Acid Reactive Substances (TBARSs)

Malondialdehyde (MDA) and other TBARSs were extracted from feeds, luminal contents, and tissues and subsequently quantified following the procedure reported by Ganhão et al.^21^ with some modifications. Samples extracted from sample homogenates were treated with 8 volumes of perchloric acid (3.86%) and 0.5 volumes of butylated hydroxytoluene (BHT) (4.2% in ethanol) to avoid further peroxidation. Upon a reaction with 0.02 M thiobarbituric acid (TBA), samples were placed in a boiling water bath (100 °C) for 45 min together with the tubes from the standard curve. After cooling, the absorbance was measured at 532 nm by spectrophotometry (Shimadzu Model UV-1800, Shimadzu, Japan). The standard curve was prepared using a 1,1,3,3-tetraethoxypropane (TEP) solution in 3.86% perchloric acid. Results were calculated as milligrams of MDA per 100 g of the sample.

#### 2.5.2 Analysis of Protein Degradation and Protein Overall Digestibility

Basal feed, intraluminal material (digests), and animal tissues from each compartment from GIT were analyzed for moisture content and concentration of protein by the official Association of Official Agricultural Chemists (AOAC) methods.^22^ The Kjeldahl method was performed as previously described by other authors.^23^ In addition to total nitrogen (TN), feed, and digests were analyzed for water-soluble nitrogen (WSN) content and nonprotein nitrogen (NPN) using the same Kjeldahl procedure. For the WSN, samples were homogenized twice with 5 volumes (w/v) of deionized water and centrifuged at 5000*g* and 4 °C for 10 min. Combined supernatants were filtered through Whatman No. 1 filter paper and subsequently subjected to the Kjeldahl method for nitrogen quantification.^22^ For the quantification of NPN, an aliquot of the aforementioned filtrate was mixed with an equal volume of 20% trichloroacetic acid (TCA), allowed to stand at room temperature for 30 min, centrifuged at 5000*g* at 4 °C for 10 min, and then filtered through Whatman No. 4 filter paper. NPN was also quantified using the Kjeldahl method.^22^ Total protein nitrogen (TPN) was calculated as follows: TPN (g) = WSN – NPN. Total dietary nitrogen (TDN) at each compartment of the GIT tract was calculated as follows: TDN (g) = (TPN – Ep) where Ep is the defined metabolic/endogenous nitrogen.^24,25^ Ep refers to nitrogen-containing biomolecules (e.g., proteins and peptides) secreted at each stage of the GIT of an animal receiving a protein-free diet. Ep was calculated for each stage and subtracted to TPN at such stage. Total dietary protein (TDP) was calculated from TDN using a conversion factor of 6.25.

An estimation of the amount of TDP degraded in each compartment of the GIT tract was calculated as follows: TDP degraded at specific compartment (g) = (TDP_1_ – TDP_2_). TDP_1_ is the total concentration of TDP in the immediately previous compartment and TDP_2_ is the concentration of TDP in the compartment under study in which digestion was assumed finished (samples taken at the end of such stage). For further accuracy, the concentration of protein in each stage was calculated considering the moisture content of feeds and luminal contents at each stage (all protein data are shown as dry matter). For the calculation of protein degradation in the stomach, TDP_1_ was considered TDP in the feeds, which corresponds to TN in the feed (×6.25), as Ep does not apply in this case for obvious reasons. The combination of TDP degraded at the stomach and at the small intestine was considered as digested protein (DP), while TDP degraded at both the cecum and the colon was considered fermented protein (FP).

An estimation of total true protein digestibility (TPD) (considering the entire GIT) was calculated according to the formula: True digestibility (%) = {[TNf – (FN – TEp)]/TNf} × 100, where TNf is total nitrogen from feeds (dietary nitrogen), FN is fecal nitrogen, and TEp is the total metabolic/endogenous nitrogen found in feces from a rat fed a protein-free diet.^25^

#### 2.5.3 Fecal Microbiota

Microbiota from *Wistar* rats was analyzed from feces obtained at slaughter, as aforementioned. DNA was isolated from feces using the MagMAX Microbiome Ultra Nucleic Acid Isolation Kit (Thermo Fisher Scientific, MA) following the manufacturer’s instructions and the KingFisher Flex Instrument (Thermo Fisher Scientific, Waltham, WA).

Genomic DNA was amplified using specific primers for V3 and V4 variable regions of the 16S rRNA gene. Amplification, sequencing, and basic analysis were performed using an Illumina MiSeq platform, using the MiSeq Reagent Kit v3 and 300b paired end. The analysis of the generated raw sequence data was carried out using QIIME2 v2021.4. Finally, the operational taxonomic units (OTUs) were classified by taxon using the SILVA database (release 138 QIIME) and trained by a scikit-learn classifier using the UNITE (release 8.3) database. Different α-diversity indices (i.e., dominance, taxa richness, individuals, Shannon index, Simpson index, and evenness) were calculated from phylum and genus OTUs’ counts using the software package Past v4.09, and the results were expressed as log_2_.

#### 2.5.4 Untargeted MS-Based Metabolomics

Metabolites were analyzed in the intraluminal colonic contents of *Wistar* rats. The extraction was carried out with both an aqueous and an organic solvent to get most of the metabolites. Briefly, 100 μL of homogenized colonic content was mixed with both 0.5 mL of cyclohexane and 0.5 mL of Milli-Q water. The mixing was homogenized in a mixer mill using small steel balls for 2 min at 30 Hz and subsequently centrifuged at 9000*g* and 4 °C for 15 min. Two phases were obtained (aqueous and organic phase) and separated into single *Eppendorf* tubes using 0.22 μm nylon filters. Additionally, 200 μL of acetonitrile HPLC quality was added to 50 μL of the aqueous phase to ensure a correct flux through the column. Samples were analyzed using a Dionex UltiMate 3000 RSLC system coupled with a Q-Exactive high-resolution mass spectrometer (Thermo Fisher Scientific, San Jose, CA). An Accucore C18 HPLC (150 × 2.1 mm^2^ I.D., particle size 2.6 μm) column was used as a stationary phase for the analysis of the organic phase, while an Accucore HILIC (150 × 3 mm^2^ I.D., particle size 2.6 μm) column was used as an aqueous phase (Thermo Fisher Scientific, San Jose, CA). The mobile phase was solvent water (eluent A) and acetonitrile (eluent B), both with 0.1% formic acid. The injection volume was 8 μL.

The gradient used for the organic phase separation was set as follows: 0–1 min isocratic 2% B, 1–14 min linear gradient 2–95% B, 14–16 min isocratic 95% B, 16–16.1 min linear gradient 95–2% B, 16.1–20 min isocratic 2% B; flow rate 400 μL/min; column temperature 45 °C; and total run time: 20 min. The gradient used for the aqueous phase separation was set as follows: 0–1 min isocratic 99% B, 1–3 min linear gradient 99–85% B, 9–10 min isocratic 5% B, 10–10.5 min linear gradient 5–99% B, 10.5–15 min isocratic 99% B; flow rate 500 μL/min; column temperature 35 °C; and total run time: 15 min. The organic phase was run under positive ionization mode, and the aqueous phase was run under both positive and negative modes.

To identify as many compounds as possible, a pool of all of the samples was run iteratively on MS^2^ analysis to achieve the mass fragmentation spectra. Full-scan analysis was used for regular samples in a scan range of 53.4–800 *m*/*z* and 70000 fwhm. MS^2^ analysis was performed for the top five data-dependent acquisitions. For both aqueous and organic LC-MS and LC-MS/MS analyses, a pool of all samples (quality control sample) was injected in every eight samples for the aligning of small shifts in retention times, mass accuracy, signal drift, and carryover, as well as normalizing peak areas if necessary. A positive identification was confirmed for discriminating metabolites by comparing MS data with those from available standard compounds. The equipment was calibrated weekly using both a Pierce LTQ Velos ESI Positive Ion Calibration Solution and a Pierce LTQ Velos ESI Negative Ion Calibration Solution (Thermo Fisher Scientific, San Jose, CA).

Data were analyzed using Compound Discoverer software (Thermo Fisher Scientific, San Jose, CA). Among the main settings used for aligning, identifying, and comparing, the metabolites found in every group had a maximum shift of 1 min and mass tolerance lower than 5 ppm.

### 2.6 Statistical Analysis

Analyses were performed in six animals per group, and each sample was technically analyzed twice. The distribution of raw data was determined by using the Shapiro–Wilk normality test. The statistical analysis of the differences among the different glycoxidative markers of the intraluminal contents and the tissues along the GIT from the two groups was carried out using a two-way ANOVA test and a Tukey test as *post hoc* analysis. The significance of differences among the protein digestibility markers and between the diversity indices was evaluated using Student-*t* tests. The data analyzed for tables and graphs by parametric tests are expressed as the mean ± standard error of the mean. Data not passing normality testing were analyzed using the Mann–Whitney U test and were expressed as the median [interquartile range (Q3 – Q1)] in the graphs. Statistical analysis was performed in SPSS version 27.0, and *p*-values lower than 0.05 were considered statistically significant. Fructose-responsive metabolites were assessed in the MetaboAnalyst (https://www.metaboanalyst.ca/), establishing standard deviation as a statistical filter for the 40% of the noninformative variables and the Pareto scaling for normalizing the raw data. Partial least-squares discriminant analysis (PLS-DA) as multivariant analysis was used, and the top 30 metabolites were ranked by the variable importance in projection (VIP) score from PLS-DA outcomes. Moreover, metabolite profile distinctions between the groups were evaluated by the Volcano plot as a one-factor statistical method to further analyze the impact of fructose on the colonic metabolome of *Wistar* rats, which combines results from fold change (FC) analysis and *t* tests into one single study using a *p*-value threshold of <0.05 and a fold change threshold >2.

## 3 Results

### 3.1 Effect of Dietary Fructose on Feed, Water, and Calories Consumption and Weights of Wistar Rats

Feed and fructose-supplemented water provided 15.31 and 5.02 kJ/g energy, respectively. Table S1 shows the median energy intake expressed as kJ/day provided by the feed, the fructose solution, and the sum of both to the experimental animals for 10 weeks. The fructose-supplemented group received significantly higher calorie intake from water consumption (*p* < 0.001). However, total energy intake per day was not significantly different. Moreover, there was no significant difference in the bodyweight of the rats during the experiment (Table S2).

### 3.2 Effect of Dietary Fructose on the Extent of Protein and Lipid Glycoxidation that Occurred in the Luminal Content of the GIT during Digestion

#### 3.2.1 Carbonylation of Digests at Different Locations of GIT

Table 1 shows the concentration of α-AS, γ-GS, and total primary protein carbonyls (sum of both α-AS and γ-GS) in the feed, digests at each stage of the GIT, and feces of the rats. Irrespective of the treatment, there were significant differences among the protein carbonylation in the feed, digests along the different gastrointestinal compartments, and in the feces (*p* < 0.001). Thus, the levels of α-AS in the digests at the stomach stage were significantly higher than those in the feed. γ-GS and total PPC showed the same trend. Overall luminal protein carbonylation increased up to 2-fold at the stomach from the experimental animals. However, the concentration of primary protein carbonyls showed a decrease in the luminal content at the jejunum stage (−30% than those in the stomach contents) (*p* < 0.001). Thereafter, the concentration of the protein glycoxidation markers displayed a progressive increase during the advance of the digest along the next stages of the GIT, the colon being the compartment where the highest concentration of carbonyls was found regardless of fructose treatment. The carbonylation level at this stage was more than 5-fold higher than that in feed. Interestingly, the concentration of both semialdehydes in the feces was 3-fold lower than in the colon stage.

**Table 1:** Concentration of Markers of Glycoxidative Stress (Means ± Standard Deviation) in the Feed, Luminal Contents (Digests) at Each Stage of the Gastrointestinal Tract, and in the Feces of *Wistar* Rats (*n* = 6 Per Group) Fed *Ad Libitum* for 10 Weeks with a Control Base Diet and either Drinking Water (Control) or a 30% Fructose Water Solution (Fructose)

		α-AS^1^	γ-GS^2^	total PPC^3^	APOPs^4^	TBARS^5^
feed		0.34^f^ ± 0.09	0.15^e^ ± 0.04	0.49^f^ ± 0.26	210^f^ ± 52	0.07^d^ ± 0.01
stomach	control	0.55^e^ ± 0.07	0.19^de^ ± 0.02	0.74^e^ ± 0.25	305^de^ ± 63	0.12^c^ ± 0.03
	fructose	0.94^cd^ ± 0.13	0.35^cd^ ± 0.06	1.29^c^ ± 0.32	541^c^ ± 48	0.11^c^ ± 0.02
jejunum	control	0.24^f^ ± 0.04	0.19d^e^ ± 0.04	0.43^f^ ± 0.12	340^d^ ± 51	0.29^a^ ± 0.06
	fructose	0.67^de^ ± 0.12	0.39^c^ ± 0.08	1.06^de^ ± 0.28	784^b^ ± 102	0.35^a^ ± 0.07
cecum	control	1.81^b^ ± 0.39	0.37^c^ ± 0.08	2.18^b^ ± 0.59	244^ef^ ± 47	0.17^b^ ± 0.02
	fructose	2.06^b^ ± 0.29	0.58^b^ ± 0.09	2.64^b^ ± 0.35	511^c^ ± 62	0.19^b^ ± 0.03
colon	control	1.17^c^ ± 0.25	0.28^d^ ± 0.07	1.45^c^ ± 0.38	366^d^ ± 43	0.15^bc^ ± 0.02
	fructose	3.25^a^ ± 0.62	1.01^a^ ± 0.18	4.26^a^ ± 0.79	1025^a^ ± 125	0.13^c^ ± 0.02
feces	control	0.45^ef^ ± 0.10	0.23^d^ ± 0.08	0.68^e^ ± 0.15	201^f^ ± 42	0.11^c^ ± 0.03
	fructose	0.86^d^ ± 0.15	0.34^cd^ ± 0.07	1.20^cd^ ±0.19	192^f^ ± 36	0.13^c^ ± 0.03
*p*-value^6^	stage	***	**	***	**	*
	diet	**	*	***	**	ns
	S × D	ns	ns	ns	*	ns

1 α-Aminoadipic semialdehyde. Results are expressed as nmol carbonyl/mg protein.

2 γ-Glutamic semialdehyde. Results are expressed as nmol carbonyl/mg protein.

3 Total primary protein carbonyls. Results are expressed as nmol carbonyl/mg of protein.

4 Advanced protein oxidation products. Results are expressed as arbitrary fluorescent units.

5 Thiobarbituric acid reactive substances. Results are expressed as mg MDA/100 g sample (feed, digests, feces).

6 Significance level in two-way ANOVA with the effects of the stage (S) (feed, GIT compartments, feces), diet (D) (control vs fructose), and the interaction (S × D).

Means with different letters within the same column were significantly different in Tukey *post hoc* analysis (*p* < 0.05). ns: no significance, *p < 0.05, **p < 0.01, and ***p < 0.001.

Fructose supplementation had a significant effect on the concentration of both α-AS and γ-GS in the luminal contents at the different stages of the GIT and in the feces of the treated animals (*p* < 0.01 and *p* < 0.05, respectively). F rats showed significantly greater amounts of total PPC in the digests at all stages than their control counterparts (*p* < 0.001). At the stomach stage, the intraluminal levels of PPC in F rats were found to be nearly doubled than those found in the stomach of control animals. At the jejunum stage, both F and C groups showed a significant decrease in the amounts of luminal PPC (*p* < 0.001). Then, the carbonyl contents in the digests at the cecum and colon stages increased, but the results showed a different trend between the groups. The amount of carbonylated proteins in the digests in the cecum from F rats was lower than those in the colon, where a significant and intense protein carbonylation occurred. Colonic digests contained the highest concentration of PPC (4.26 nmol carbonyls/mg of protein), being more than 8-fold higher than that found in feeds (*p* < 0.001). Instead, the highest concentration of semialdehydes in the digests from the C group occurred in the cecum. The concentration of carbonyls in the feces from the F group was significantly lower than in the feces from C rats. The interaction between fructose supplementation and the effect of the different stages of GIT was not statistically significant, meaning that the effect of fructose is location-independent.

#### 3.2.2 Formation of APOPs in Digests at Different Locations of GIT

In addition to the glycoxidation markers described above, Table 1 shows the evolution of the amounts of APOPs in the digests along the different GIT stages as markers of advanced protein glycation processes. The intensity of the fluorescence emitted by APOPs significantly showed 2.7-fold higher values from feed to digests at the jejunum stage (*p* < 0.01). Then, the presence of these compounds reached the highest values in the intraluminal contents at the colon stage, while it diminished in the feces. Fructose treatment significantly enhanced the formation of APOPs in the luminal contents from the GIT (*p* < 0.01), except in feces. The colonic contents from F rats showed 1.5-fold higher values of fluorescent units due to the presence of APOPs than their C counterparts. Fructose enhanced the formation of APOPs at all digestion stages and samples except in the feces.

#### 3.2.3 Lipid Oxidation in Digests at Different Locations of GIT

Table 1 also shows the extent of lipid oxidation expressed as amounts of TBARS (mg of MDA/100 g sample). Lipid oxidation significantly increased up to 4.5-fold in the jejunal contents from the rats after basal diet ingestion (i.e., mean values of 0.32 mg MDA/100 g sample) (*p* < 0.05). These highest mean values significantly decreased at the next stages of digestion until mean values of 0.12 mg MDA/100 g sample in the feces of the animals. Fructose treatment did not have any significant effects on the extent of lipid oxidation.

### 3.3 Effect of Dietary Fructose on Glycoxidative Stress in Tissues from GIT

Table 2 shows the concentration of individual carbonyls and total PPC in the tissues from each compartment of the GIT from *Wistar* rats. The levels of total PPC in the tissues significantly increased through the different GIT stages regardless of the treatment with fructose, reaching more than 2-fold higher PPC at the jejunum stage from the experimental animals than that found in the stomach tissue (*p* < 0.01). The fructose treatment significantly increased the amounts of semialdehydes in both, the stomach and jejunum tissues (*p* < 0.01). At the colonic stage, the concentration of the glycoxidative markers in the tissue increased significantly in animals subjected to fructose supplementation. Fructose intake significantly enhanced the formation of APOPs in all tissues of the GIT (*p* < 0.01). Moreover, the values of APOPs in the colon from the F group significantly peaked at 1203 FU. Meanwhile, lipid oxidation showed some variations among GIT tissues, and fructose consumption did not have any significant effect on these values.

**Table 2:** Concentration of Markers of Glycoxidative Stress (Means ± Standard Deviation) in the Tissues from Each Compartment of the Gastrointestinal Tract from *Wistar* Rats (*n* = 6 Per Group) Fed *Ad Libitum* for 10 Weeks with a Control Base Diet and either Drinking Water (Control) or a 30% Fructose Water Solution (Fructose)

		α-AS^1^	γ-GS^2^	total PPC^3^	APOPs^4^	TBARS^5^
stomach	control	0.38^e^ ± 0.09	0.22^c^ ± 0.02	0.61^d^ ± 0.14	350^f^ ± 22	0.26^b^ ± 0.06
	fructose	0.69^d^ ± 0.12	0.46^b^ ± 0.05	1.15^c^ ± 0.22	506^d^ ± 31	0.29^b^ ± 0.04
jejunum	control	1.29^c^ ± 0.16	0.50^a^ ± 0.07	1.80^b^ ± 0.25	439^e^ ± 30	0.38^a^ ± 0.06
	fructose	1.38^c^ ± 0.19	0.56^a^ ± 0.06	1.95^b^ ± 0.31	627^c^ ± 44	0.41^a^ ± 0.09
colon	control	1.99^b^ ± 0.25	0.12^d^ ± 0.03	2.03^b^ ± 0.29	840^b^ ± 87	0.36^ab^ ± 0.07
	fructose	2.45^a^ ± 0.29	0.15^d^ ± 0.04	2.61^a^ ± 0.32	1203^a^ ± 99	0.41^a^ ± 0.11
*p*-value^6^	stage	***	***	**	***	*
	diet	***	**	**	***	ns
	S × D	*	**	*	ns	ns

1 α-Aminoadipic semialdehyde. Results are expressed as nmol carbonyl/mg protein.

2 γ-Glutamic semialdehyde. Results are expressed as nmol carbonyl/mg protein.

3 Total primary protein carbonyls. Results are expressed as nmol carbonyl/mg of protein.

4 Advanced protein oxidation products. Results are expressed as arbitrary fluorescent units.

5 Thiobarbituric acid reactive substances. Results are expressed as mg MDA/100 g sample (feed, digests, feces).

6 Significance level in two-way ANOVA with the effects of the stage (S) (feed, GIT compartments, feces), diet (D) (control vs fructose), and the interaction (S × D).

Means with different letters within the same column were significantly different in Tukey *post hoc* analysis (*p* < 0.05). ns: no significance, *p < 0.05, **p < 0.01, and ***p < 0.001.

### 3.4 Effect of Dietary Fructose on Protein Digestion

#### 3.4.1 Protein Degradation during Digestion

**Figure 1:**
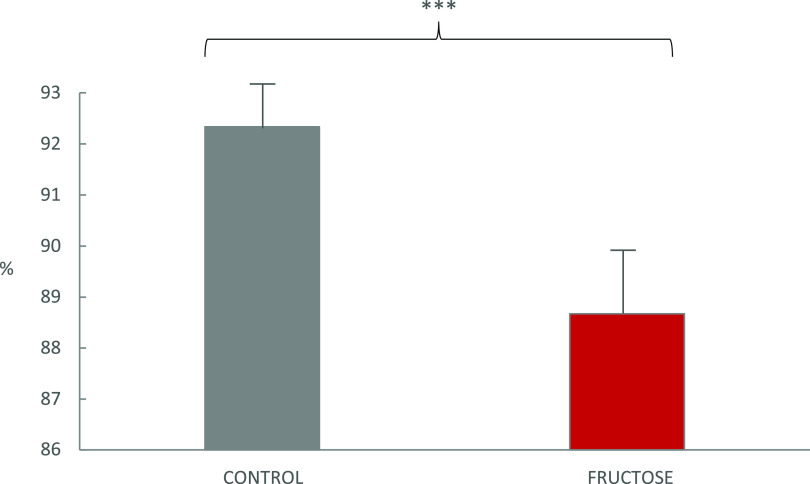
True protein digestibility^a^ of a basal feed (∼15% crude protein dry matter) in *Wistar rats* as affected by either drinking water (control) or a 30% fructose water solution for 10 weeks. ^a^True protein digestibility (%) = {[TNf – (FN – TEp)]/TNf} × 100, where TNf is total nitrogen from feeds (dietary nitrogen), FN is fecal nitrogen, and TEp is the total metabolic/endogenous nitrogen found in feces from a rat fed a protein-free diet.^25^ The pair of means with asterisks is significantly different in Student-*t* tests: ***p < 0.001.

**Figure 2:**
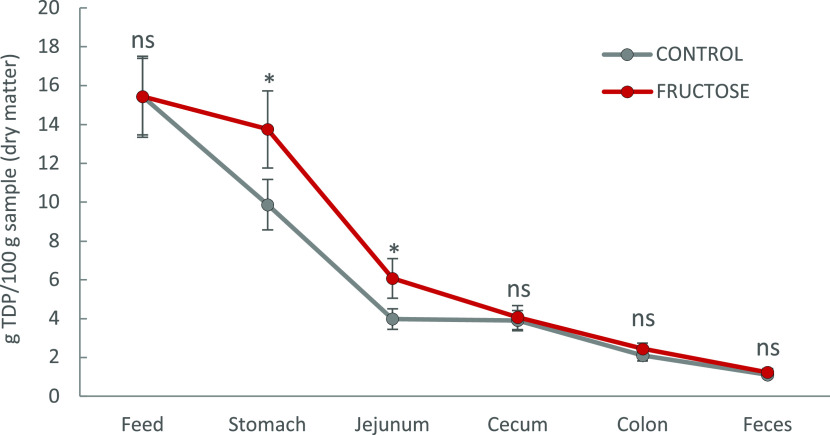
Evolution of the concentration of total dietary protein (TDP)^a^ at the different stages of the *in vivo* digestion of basal feed (∼15% crude protein dry matter) as affected by either drinking water (control) or a 30% fructose water solution for 10 weeks. ^a^Total dietary protein (TDP, g/100 g digests) was calculated in feeds, luminal material of each compartment of the GIT, and feces as follows: TDP (g)= [(WSN – NPN) – Ep] × 6.25; WSN is water-soluble nitrogen, NPN is nonprotein nitrogen, and Ep is the metabolic/endogenous nitrogen.^24,25^ Ep refers to nitrogen-containing biomolecules (i.e., proteins, peptides, etc.) secreted at each stage of the GIT of an animal receiving a protein-free diet. Results are presented in dry matter, and hence, the moisture of each sample was also taken into account.

**Figure 3:**
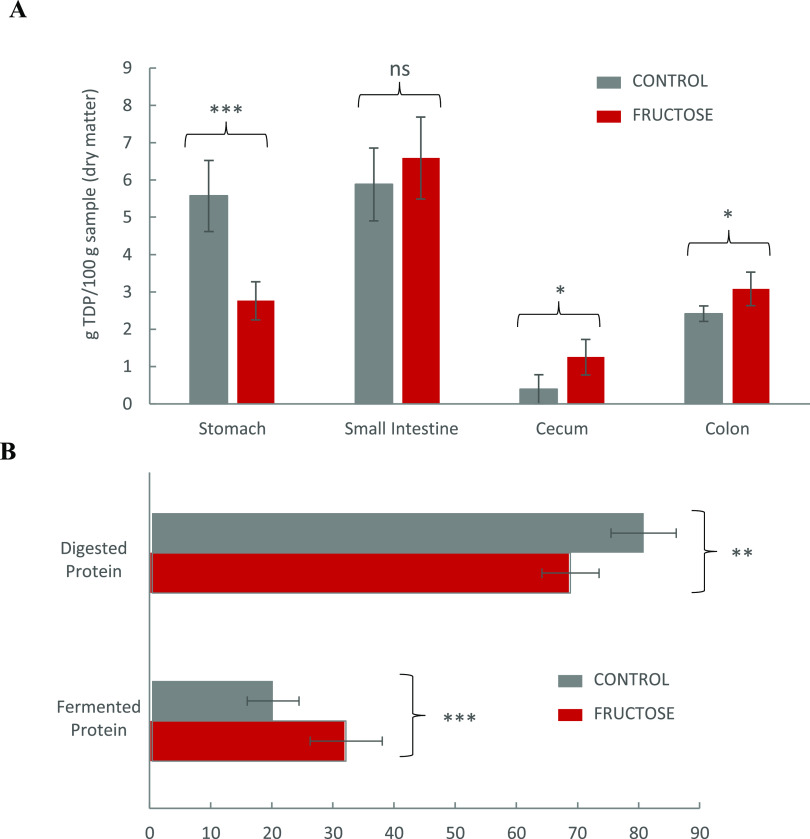
(A) Amount of total dietary protein (TDP) degraded^a^ at the different compartments of the GIT of from *Wistar* rats (*n* = 6 per group) fed *ad libitum* for 10 weeks with a control base diet and either drinking water (control) or a 30% fructose water solution (fructose). (B) Percentage of TDP digested (degraded in stomach + small intestine) vs percentage of TDP fermented (degraded in cecum + colon) in control and fructose groups. ^a^Degraded TDP at each specific compartment (g) was calculated as (TDP_1_ – TDP_2_); where TDP_1_ is the total concentration of TDP in the immediately previous compartment and TDP_2_ is the concentration of TDP in the compartment under study in which digestion was assumed finished (samples taken at the end of such stage). For further accuracy, the concentration of protein in each stage was calculated considering the moisture content of feeds and luminal contents at each stage (all protein data are shown as dry matter). For the calculation of protein degradation in the stomach, TDP_1_ was considered TDP in the feeds, which corresponds to TP in the feed (TN × 6.25), as Ep does not apply in this case for obvious reasons. The pair of means with asterisks is significantly different in Student-*t* tests. *p < 0.05, **p < 0.01, ***p < 0.001, and ns: no significant differences.

Figure 1 shows that the TPD of the basal diet provided to *Wistar* rats significantly decreased in rats exposed to fructose as compared to C rats (88.7% vs 92.3%, respectively; *p* < 0.001). To comprehend underlying mechanisms, an in-depth study of protein digestion was carried out. Figure 2 shows the evolution of TDP from the feed, during the different digestion stages at the GIT, and in the feces of experimental animals. TDP decreased as the digests advanced through the compartments of the GIT of the animals regardless of the fructose supplementation. However, the trend of dietary protein degradation was different when fructose was consumed by the rats. In fact, we analyzed the extent of protein degradation at each compartment as TDP degraded. Figure 3A shows the amount of degraded TDP at the different compartments of the GIT from C and F *Wistar* rats. Figure 3B shows the percentage of proteins that were degraded in the stomach and jejunum (“digested proteins”), and the percentage of proteins that were degraded at the cecum and colon stages (“fermented proteins”). Irrespective of the treatment, the highest rates of protein degradation were found at the initial stages of digestion. Yet, when fructose was supplied to animals, the digestion of TDP in the stomach was significantly reduced to less than half of the TDP digested in the stomach of C rats. Overall, 80% of dietary proteins were digested (stomach and jejunum) in GIT of C rats, while only 68% of dietary proteins was digested in rats drinking fructose (*p* < 0.01). Conversely, around 32% of TDP was fermented (cecum and colon) in rats drinking fructose, while a significantly lower protein percentage (20%) was fermented at the same stages in C animals (*p* < 0.001).

### 3.5 Effect of Dietary Fructose on Microbiota

To elucidate possible changes in the gut microbiome of *Wistar* rats after the high intake of fructose for 10 weeks, we analyzed the different α-diversity indices at the phylum and genus levels from the different OTU counts obtained. There were no significant differences between C and F rats in either the values of the diversity indices or the relative abundance of taxa at the phylum level (data are not shown). However, at the genus level, the microbiota of F rats contained significantly higher amounts of individuals than the microbiota of C rats (*p* < 0.05) (Table 3). In fact, specific genera were found only in the fecal microbiota from F rats.

**Table 3:** α-Diversity Index Values Expressed as Log_2_-Means ± Standard Error of the Mean at the Genus Level from the Fecal Microbiome of *Wistar* Rats (*n* = 6 Per Group) Fed *Ad Libitum* for 10 Weeks with a Control Base Diet and Either Drinking Water (control) or a 30% Fructose Water Solution (Fructose)

	taxa richness	individuals	dominance	Simpson index	Shannon index	evenness
fructose	62.83 ± 1.80	27 917.00 ± 14 179.10	0.19 ± 0.01	0.81 ± 0.01	3.31 ± 0.09	0.16 ± 0.01
control	58.83 ± 2.32	22 663.83 ± 1680.78	0.21 ± 0.02	0.79 ± 0.02	3.18 ± 0.16	0.16 ± 0.01
*p*-value^a^	ns	*	ns	ns	ns	ns

a Significance level in the Student-*t* test with the effects of the diet (fructose and control). *p < 0.05 and ns: not significant.

Nevertheless, significant changes in the relative abundance of some microorganisms at the genus level were observed (Supporting Information). Although its different occurrence did not alter the α-diversity index, these changes may be remarkable and deserve attention. Thus, the microbiome of F rats was characterized by significantly higher amounts of *Christensenellaceae R-7 group* species, uncultured *Lachnospiraceae* spp., *Clostridia vadin* BB60 group spp. and uncultured *Ruminococcaceae* spp. Meanwhile, *Lactobacillus* spp., *Egerthellaceae DNF00809* spp., and *Bifidobacterium* spp. were significantly lower expressed in the F group than in their control counterparts. Species of the *Eubacterium nodatum* group from the *Anaerovoraceae* family and *Adlercreutzia* spp. were found only in the fructose group. *Desulfovibrio* spp. and genera of the family *Oscillospirales UCG-10* showed an increased trend in the F group, while *Streptococcus* spp. diminished (0.05 < *p* < 0.1).

Moreover, a range of species from selected genera proposed as fructose-sensitive and/or proteolytic was analyzed (Table S3). Long-term fructose intake significantly decreased the relative abundance of *Bifidobacterium animalis* (*p* < 0.05). In addition, *Alistipes shashii* (*p* < 0.05) occurred only in the microbiome of F rats. Moreover, some trends were remarkable in relation to the impact of fructose on the microbiota of F rats (*p*-values = 0.05), such as a lower relative abundance of *Lactobacillus grasseri* and an unclassified bacterium from genera *Streptococcus*, as well as the higher expression of an uncultured bacterium from genera *Marvinbryantia*.

### 3.6 Effect of Dietary Fructose on Colonic Metabolome from Wistar Rats

The untargeted metabolomic analysis revealed 2317 metabolites in the intraluminal contents of the colon from C and F *Wistar* rats. Compound Discoverer software paired the compounds name and/or formula with the calculated weights of the detected molecules using different databases (i.e., AKos, BioCyc, Chemspace, FooDB, Human Metabolome Database, KEGG, LipidMAPS, Mcule, Nature Chemical Biology, Nature Chemistry, NPAtlas, Toxin, Toxin-Target Database and Urine Metabolome Database). According to the routine calibration and optimization of the equipment, as well as our metabolite extraction method, the identification and characterization of the metabolites (Table S4) belong to level 2 of the identification levels proposed by the published metabolomics literature.^26^

**Figure 4:**
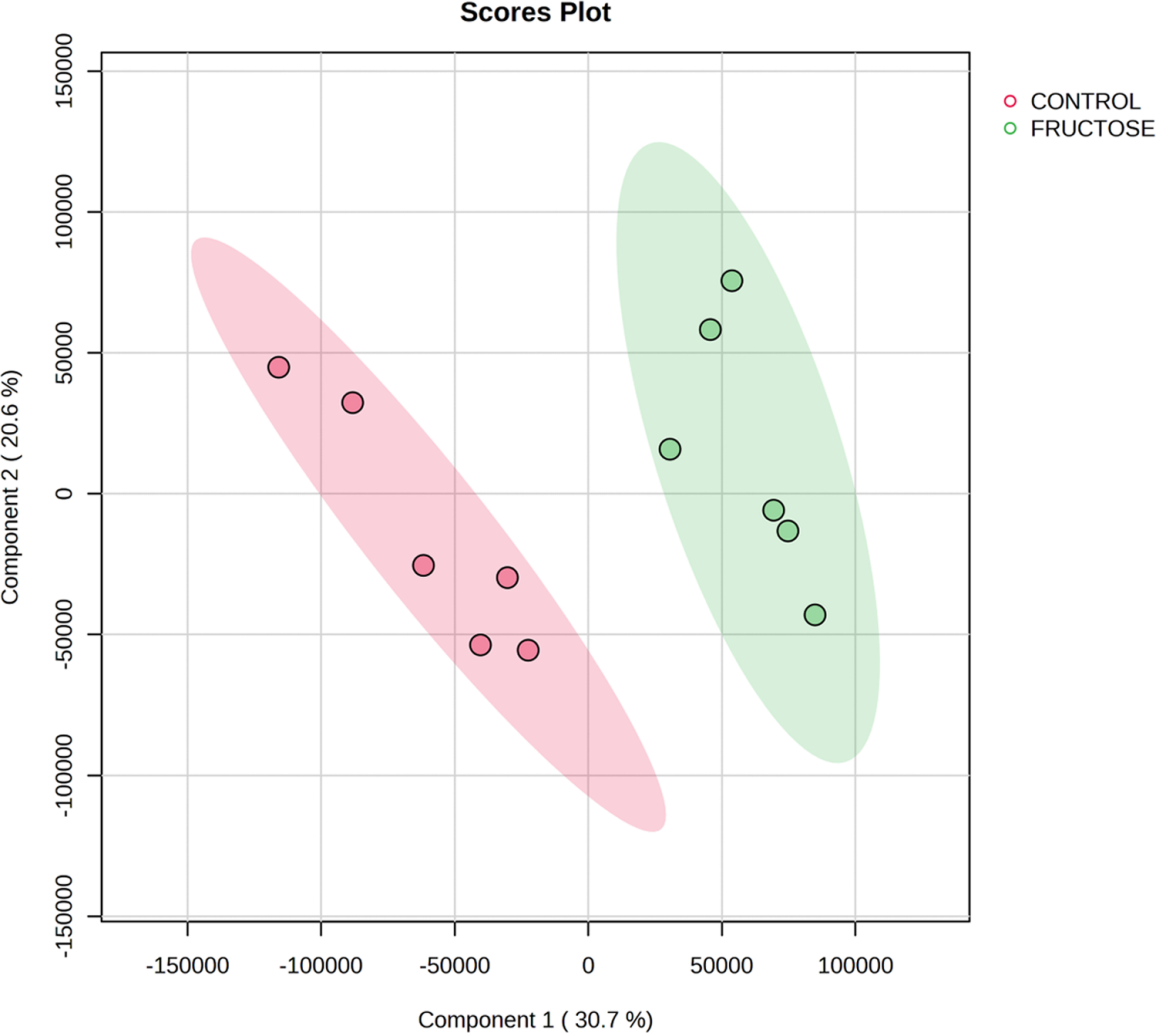
Score plots from the partial least-squares discriminant analysis multivariant analysis of the colonic contents from *Wistar* rats (*n* = 6 per group) fed *ad libitum* for 10 weeks with a control base diet and either drinking water (control) or a 30% fructose water solution (fructose).

**Figure 5:**
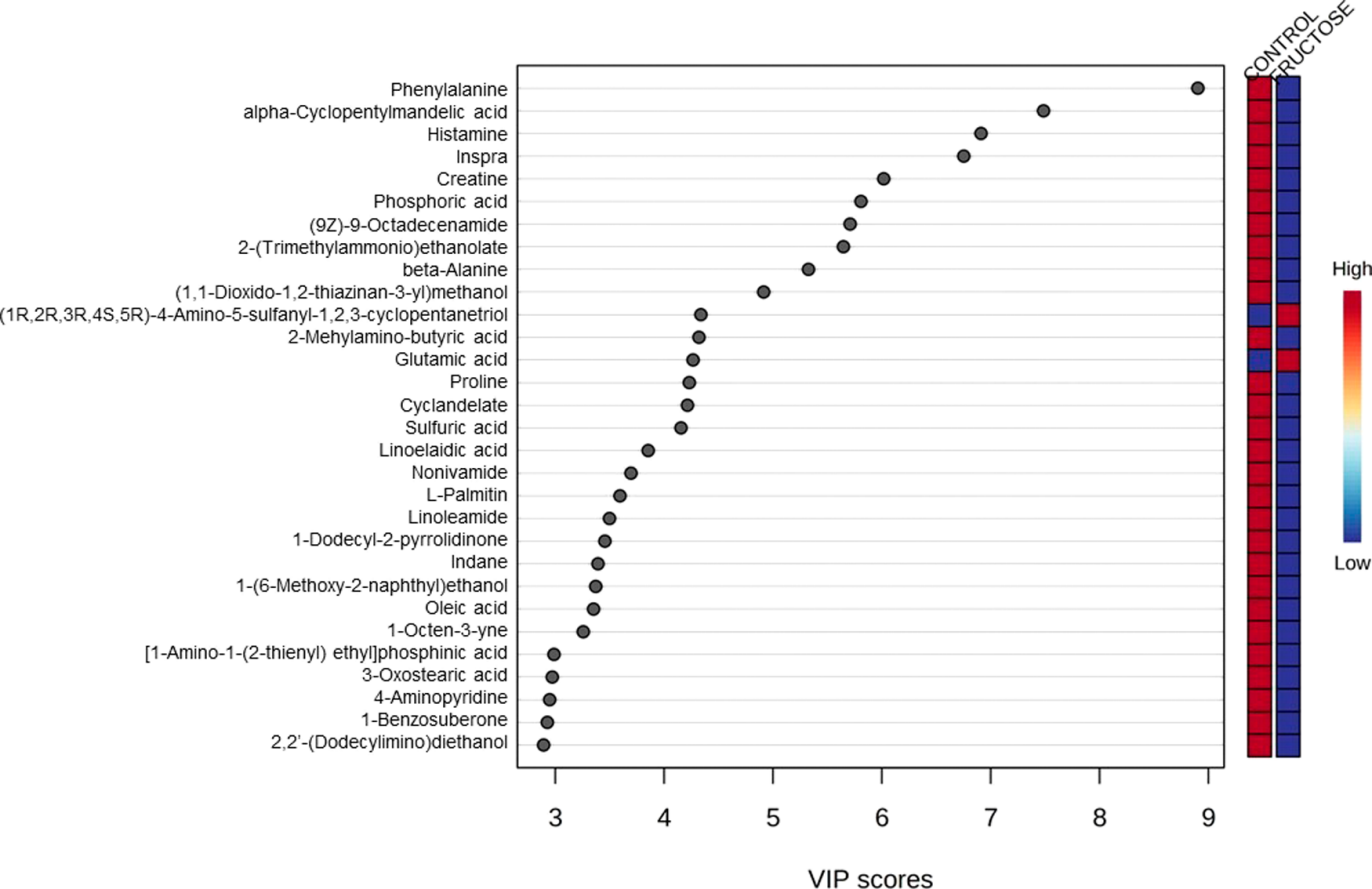
Variable importance in projection (VIP) score plot multivariant analysis outcomes from metabolomic results of the colonic contents from *Wistar* rats (*n* = 6 per group) fed *ad libitum* for 10 weeks with a control base diet and either drinking water (control) or a 30% fructose water solution (fructose).

Overall, 385 metabolites were only detected in the colonic contents of the C rats, while 520 were only found in the colonic digests of F rats. In order to analyze the results, the peak intensities of the metabolites were compared using Metaboanalyst software (https://www.metaboanalyst.ca/). According to the PLSD-DA plot, a different clustering of colonic contents was observed due to the fructose treatment (Figure 4). The VIP score is an important measure that estimates the importance of each variable in the projection used in a PLS-DA model. Figure 5 shows the main loadings inferred by the analysis, with the relative concentrations of the corresponding metabolite in each group under study in the colored boxes on the right.

Fructose intake increased the concentration of 196 metabolites and decreased the concentration of 486 metabolites as compared to that of colonic digests from C rats. In particular, fructose promoted a higher abundance of some relevant metabolites such as β-d-glucose 6-phosphate (fold change: 7.56; *p*-value: 0.03), 2-aminobutanoic acid (fold change: 6.90; *p*-value: 0.001), cadaverine (fold change: 6.27; *p*-value: 0.006), prolylleucylglycine (fold change: 4.26; *p*-value: 0.002), serylglycine (fold change: 3.32; *p*-value <0.001), pyruvic acid (fold change: 2.67; *p*-value <0.001), lactic acid (fold change: 2.24; *p*-value <0.001), tryptophan (fold change: 1.10; *p*-value: 0.02), and 2-oxobutyric acid (fold change: 1.09; *p*-value <0.001), among several others (volcano, Supporting Information). On the other hand, fructose intake reduced the quantity of several metabolites, such as β-alanine (fold change: −5.57; *p*-value <0.001), spermidine (fold change: −3.70; *p*-value <0.001), hypotaurine (fold change: −3.24; *p*-value <0.001), acetic acid (fold change: −2.76; *p*-value <0.001), 2,6-diaminopimelic acid (fold change: −2.64; *p*-value <0.001), maleic acid (fold change: −2.61; *p*-value <0.001), glyceraldehyde (fold change: −2.54; *p*-value <0.001), γ-aminobutyric acid (GABA) (fold change: −2.21; *p*-value: 0.003), glycerol 3-phosphate (fold change: −1.87; *p*-value:0.02), dihydroxyphenylalanine (l-dopa) (fold change: −1.83; *p*-value: 0.02), and histamine (fold change: −1.63; *p*-value: 0.004), among others (volcano, Supporting Information).

**Figure 6:**
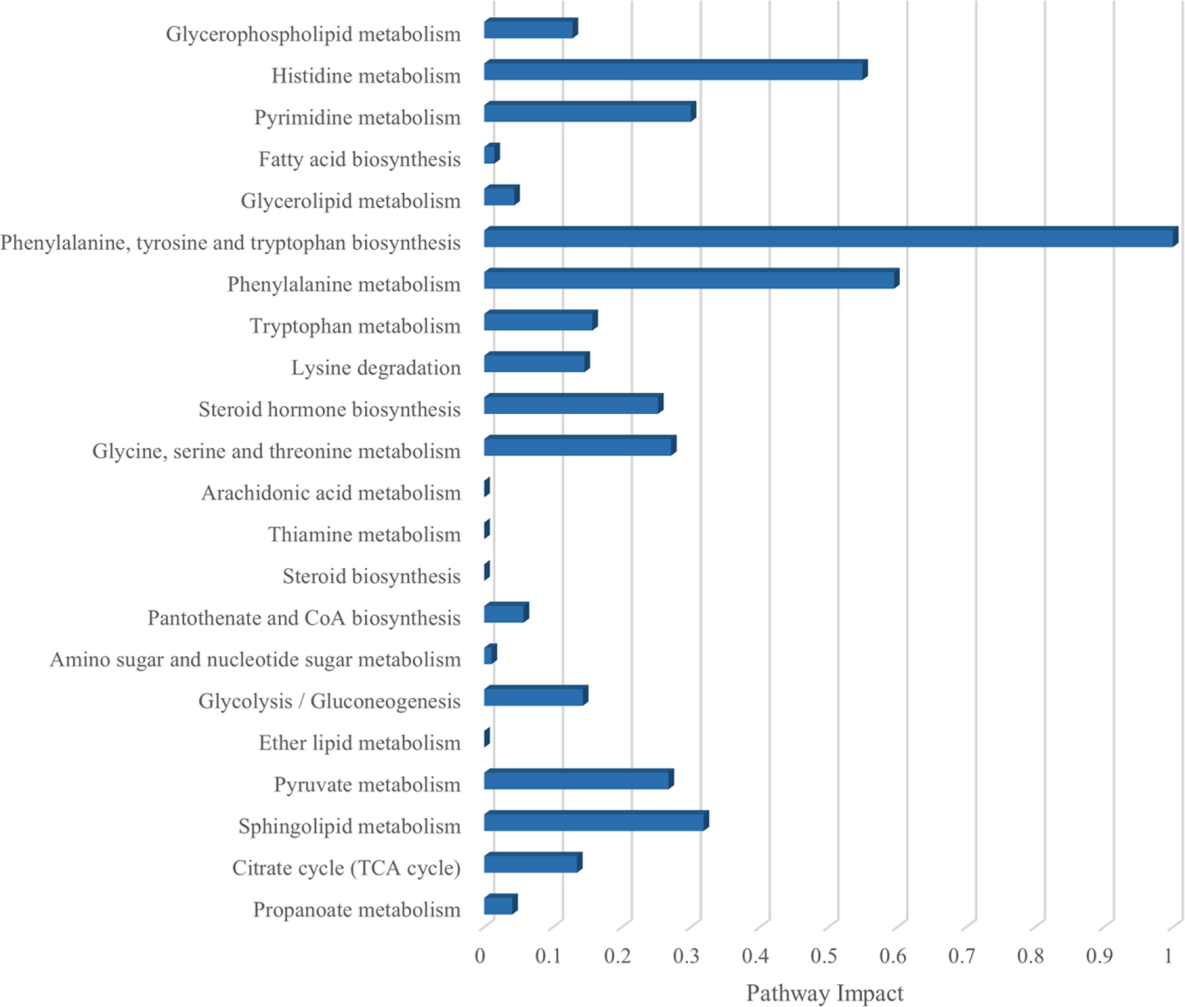
Categorization of the main pathways resulting from the pathway enrichment analysis (*p* < 0.005) and the pathway impact values according to the pathway topology analysis, highlighted from the metabolomics analysis of the data from the colonic contents from treated *Wistar* rats (*n* = 6 per group), fed *ad libitum* for 10 weeks with a control base diet and a 30% fructose water solution (fructose) regarding control water-consumer counterparts.

Based on the categorical differential metabolites, a pathway enrichment analysis and KEGG topology analyses were performed in Metaboanalyst software (https://www.metaboanalyst.ca/) to evaluate metabolic changes induced by the long-term intake of fructose. The categorization of the results was carried out according to the *p*-values from the pathway enrichment analysis and the pathway impact values from the topology analysis. Thus, 22 metabolic pathways were significantly affected by the fructose treatment in the intraluminal colonic content of the rats. Figure 6 shows the significant pathways affected by the treatment after the enrichment analysis, including pathways involved in energy metabolism as glycolysis, citrate cycle, or pyruvate metabolism (*p* < 0.001, respectively) and pathways related to the metabolism of certain amino acids as histidine, phenylalanine, tryptophan, and lysine (*p* < 0.05).

## 4 Discussion

To the best of our knowledge, this study provides the first assessment of the impact of sustained consumption (10 weeks) of fructose on the intraluminal (digests) and tissue oxidative stress from different compartments of the GIT from *Wistar* rats. Our results are novel in highlighting the molecular mechanisms behind the potential reactivity of fructose with dietary proteins and other components from GIT that could be the basis of the undesirable effects of the consumption of this reducing sugar on tissues and peripheral organs.

### 4.1 Glycoxidative Stress in the Lumen of GIT and Impaired Digestibility

Dietary protein digestion begins once it reaches the stomach. The gastric juices promote the unfolding of proteins, ensuring the recognition and action of gastric enzymes.^27^ However, protein denaturation could also enhance the exposure of hydrophobic groups in proteins, which, along with protein oxidation, facilitates protein cross-linking and aggregation.^28^ In fact, it has been documented that the pro-oxidative environment of the stomach promotes the oxidation of proteins in several *in vitro*(15,29,30) and *in vivo* studies,^31^ which is in agreement with our results. The ability of reducing sugars to induce the onset of glycoxidative reactions in dietary proteins has been documented by a few *in vitro* studies in which the physiological conditions of the stomach were simulated.^15,32,33^ The present *in vivo* study confirms that dietary fructose promotes the creation of a severe pro-oxidative environment in the stomach of *Wistar* rats, stimulating the oxidative damage of dietary proteins. Fructose has been profusely studied in relation to the mechanisms implicated in such glycoxidative stress. One of these mechanisms is the ability of fructose to generate RCS (i.e., glyoxal and methylglyoxal), either by products of its autoxidation (“Wolf pathway”) or by its role in Maillard reactions (“fructosylation”).^34^ Moreover, fructose has long been described as much more reactive than glucose in Maillard reactions due to the stability of its open-chain form and its keto group.^34,35^ The glycoxidation of proteins involves the reaction of susceptible protein residues with RCS.^6,8^ RCS triggers the deamination of protein-bound alkaline amino acids, which leads to the formation of primary protein carbonyls, such as α-AS, derived from lysine, and γ-GS, derived from arginine and proline.^36^ These semialdehydes represent the most abundant carbonyls formed during protein glycoxidation,^37^ so both individual detection and quantification are relevant as expressions of the levels of glycoxidative stress. Accordingly, our results indicate that the intake of a high-fructose (30%) solution for 10 weeks significantly promotes the *in vivo* formation of PPC in the stomach contents of Wistar rats (*p* < 0.001). On the other hand, at the first steps of fructosylation, a covalent interaction between the free carbonyl group of open-chain fructose and the amino group of proteins could occur and generate Schiff bases, which would lead to the formation of Heyns products by several chains of reactions. It is believed that the Heyns products, RCS, and reactive oxygen species (ROS) formed during fructosylation are important precursors of nonenzymatic adducts of the proteins as APOPs (i.e., AGES). Each protein fructosylation reaction releases a superoxide radical, so fructose generates 100 times more ROS than glucose and promotes cell apoptosis and inflammation.^38^ Since α-AS and γ-GS are also formed in proteins as the direct electrophilic attack of ROS,^39^ it is impossible to state the extent to which RCS and/or ROS contributed to the carbonylation of dietary proteins. It is, yet, indisputable that fructose effectively contributes to creating a pro-oxidative environment in the stomach, as previously stated for glucose in an *in vitro* study.^15^ Up to now, there was *in vitro* evidence of fructose inducing formation of APOPs at the first stages of the GIT.^32,33^ This study confirms for the first time that such reactions also occur in an *in vivo* gastrointestinal system. Our results revealed the harmful reactivity of fructose with dietary proteins during *in vivo* gastric digestion and the lack of effect of glycoxidative reactions on dietary lipids. These results are in agreement with previous reports in which proteins seemed to be the most relevant target of oxidative reactions during both *in vitro* and *in vivo* digestion of various muscle foods.^31,40^

The increased protein glycoxidation caused by the intake of 30% of fructose in the stomach seemed to affect the digestion pattern of proteins, which would remain undigested in the lumen of the next stages of the GIT. Thus, this is reflected in the higher values of the glycoxidative stress markers analyzed in the digests at the jejunum stage of F-treated rats as compared to C ones. The small intestine has many more specific proteolytic enzymes than the stomach.^41^ The resulting di- and tripeptides and single amino acids from enzymatic digestion can be absorbed into the bloodstream, as well as the carbonylated residues, and this could be the reason for the significant decrease in PPC in the jejunum digests from the F group. Likewise, the amount of TDP decreased in the jejunal contents, and the protein degradation reached the highest values at this intestinal compartment, as expected. Unlike what was found in the stomach, fructose administration had no effect on the degradation of dietary proteins in the small intestine (*p* > 0.05). It is worth highlighting that the degree of protein digestion in C rats was similar in the stomach and small intestine (5–6 g of TDP digested in each stage). The amount of TDP digested in the small intestine of rats treated with fructose was remarkably more abundant than that digested in the stomach (6.7 g vs 2.6 g). It is hence reasonable to hypothesize that the impaired digestion caused by fructose in the stomach was partially counteracted by more intense protein digestion in the small intestine. Yet, the total digested protein (stomach + small intestine) was significantly lower in animals exposed to dietary fructose. Severe protein glycoxidation impairs protein digestibility by modifying the amino acid composition (carbonylation) and reasonably altering the accessibility and recognition of proteolytic enzymes to the cleavage site.^42−44^ These results confirm previous findings in which glucose-mediated protein carbonylation during simulated digestion of meat and dairy proteins led to an impaired digestibility of such proteins.^15,36^ Therefore, the amount of undigested and presumably glycoxylated proteins reaching distant locations of the GIT was significantly higher in rats exposed to fructose.

The lack of degradation of glycoxylated proteins in the first compartments of the GIT could have facilitated their arrival to the cecum and colon, where they were eventually fermented by gut microbiota.^45^ In fact, the depletion of TDP in the cecum/colon, attributed to the degradation of proteins by microbiota, was significantly higher in rats fed with fructose than in the C counterparts. The occurrence of oxidative and glycoxidative reactions at this stage is of particular clinical interest, given that most functional and organic disorders diagnosed in human GIT are located in the colon.^46^ It is, therefore, highly meaningful that the concentration of all protein glycoxidation markers (PPC and APOPs) peaked in the colonic lumen and tissue of rats provided with dietary fructose. In addition to the arrival to this stage of glycoxylated proteins from previous stages, there was a net increase of all protein oxidation markers in the colon. The remarkable buildup of PPC, glycoxylated proteins, and AGES in the intraluminal contents at the colon stage shows the relevance of this GIT compartment as a truly redox-active environment where both the oxidation of dietary components and microbiota interact.^47^ The fact that fructose-exposed rats suffered more intense glycoxidative reactions at this stage may imply that fructose and/or their reactive degradation products reached this distant location of the GIT as well as nondigested glycoxylated proteins, which would promote the onset of further oxidative reactions in the colon. In this regard, a timely connection at this stage of redox reactions and inflammatory processes has been described since chronic oxidation would lead to proinflammatory pathways, and inflammation, itself, contributes to the onset of a pro-oxidative environment.^47^ The role of dietary AGES in gut inflammation and gut microbial composition was deciphered.^48^ While the occurrence of dietary fructose/RCS at this stage cannot be ruled out, the products of its protein glycosylation reactions may be implicated more likely in the promotion of luminal and tissue oxidative stress in the colon. It is common knowledge that the transformation of undigested compounds either by the host or by the microbiota increases the rate of oxidative stress and the formation of several metabolites in the luminal content of the GIT.^47^ An increased pro-oxidative environment and a greater amount of undigested protein owing to a previously impaired digestibility would facilitate the microbiome degradation of this luminal material to the production of potentially toxic metabolites.^49^ The identification of some of these microbial metabolites is of enormous scientific interest since it is reported that certain protein fermentation products in the colon can be proinflammatory and carcinogenic.^49,50^ The highest uptake of nitrogen at these stages in rats exposed to fructose may have relevant pathophysiological consequences, given that most of that nitrogen compounds would have resulted from microbiota fermentation of at least partially oxidized proteins.

### 4.2 Glycoxidative Stress in Tissues of GIT

It is well documented that increased glycoxidative stress in the lumen of the gastrointestinal tract contributes to the damage of neighboring tissues.^51,52^ It is therefore reasonable that the stomach tissue from rats provided with fructose had higher rates of protein glycoxidation markers (PPC and APOPs) than their C counterparts. Therefore, the onset of intraluminal glycoxidative stress in the stomach could have promoted *in situ* protein glycoxidation of the tissue. In addition to the potential uptake of oxidized species at this stage, the absorption of reactive fructose and RCS derived from its degradation could have promoted oxidative damage in proteins from the stomach tissue. Numerous gastroduodenal diseases are related to increased inflammatory processes derived from ROS attacks, such as peptic ulcer, gastritis, or gastric cancer.^53^ More specifically, protein oxidation was emphasized as the most salient biochemical process in patients suffering from *Helicobacter pylori* chronic infection and gastric cancer.^54^ Moreover, these authors displayed that the extent of lipid oxidation was not a reliable marker of the disease, even though it decreased in cancer patients as compared to healthy individuals. This is in line with the current results, in which lipid oxidation was negligible as compared to the oxidative damage to proteins. Carbonylation levels in mucosa from healthy individuals are around 1–2 nmol protein hydrazones/mg protein,^55^ while above 2 nmol protein hydrazones/mg protein was reported in plasma from gastric cancer patients.^54^ It is crucial to highlight that the aforementioned authors quantified total protein carbonyls using the routine spectrophotometric dinitrophenylhydrazine method, which is well known for overestimating the concentration of primary protein carbonyls in biological samples.^8^ Taking into account that the sum of α-AS and γ-GS account for between 50 and 70% of protein hydrazones,^8,37^ the concentration of PPC found in the stomach tissue of rats subjected to sustained consumption of fructose may be within the pathological range. The lack of information on specific protein carbonyls in pathological conditions affects the comprehension of the role of protein carbonylation in human diseases.^56^

The extent of protein glycoxidation in the jejunal tissue from F rats was higher than that in the previous compartment (stomach). The accretion of oxidation products, such as protein carbonyls in the epithelium of the intestinal mucosa, as a first stage of their intestinal uptake and bloodstream distribution to internal organs was hypothesized.^52^ This, in fact, could explain the depletion of carbonylated proteins in the luminal content at the intestinal stage under study and, consequently, the increased carbonylation in the jejunal tissue. Additionally, fructose and related RCS may have been uptake and induce, *in situ*, carbonylation of tissue proteins at this location as well. Some authors have carried out *in vivo* experiments aiming to evaluate the levels of oxidative stress in the tissue of the small intestine by different markers when high amounts of fructose are ingested.^57^ In line with the present results, the authors found increased concentrations of various markers of oxidative and nitroxidative stress in proteins from the small intestine of rodents that were exposed to a 30% fructose drinking water solution for 8 weeks.^57^ Fructose-exposed mice suffered intestinal barrier dysfunction and endotoxemia along with liver fibrosis.^11^ How fructose contributes to the disintegration of intestinal tight junction proteins, which may facilitate the subsequent uptake of intestinal toxins, was comprehensively illustrated in a previous study.^58^ Further to the role of PPC in intestinal function and health, it is also involved in the formation of advanced glycation and oxidation products such as AGES/APOPs.^59^ The involvement of PPC in such reactions could explain its depletion in the jejunal lumen and the increased amounts of APOPs at the same location, particularly in fructose-exposed rats (*p* < 0.001). Some authors reported that the formation of intestinal AGES from the reaction of dietary fructose with peptides and amino acids might be the triggering point of the inflammatory bowel response associated with high fructose intake.^32^ Consistently, in our study, the jejunal tissue from rats supplemented with fructose showed higher amounts of APOPs than C rats (*p* < 0.05), which could be secondary to the uptake of luminal glycoxidation products or formed *in situ*, subsequent to the uptake of reactive carbonyls.

Diet-derived AGES has been demonstrated to interfere with many cell functions such as lipid synthesis, inflammation, antioxidant defenses, and mitochondrial metabolism due to its accretion in target tissues,^10^ but this is the first study that analyzed the endogenous formation of AGES and its plausible accretion in the tissues from GIT stages in an *in vivo* experiment. Oral administrated fructose is mainly cleared by the small intestine, where it is converted into glucose and organic acid.^60^ Hence, the small intestine exerts a great influence on the consequent metabolic disorders associated with excessive fructose intake.^58^ Intestinal metabolism of fructose is ATP-dependent, which could increase the protein carbonylation in the tissue at the stage by the increased secondary-ROS production.^3,60,61^ When high amounts of fructose are ingested, changes in the energy homeostasis are manifested and oxidative stress and intestinal inflammatory response are induced, disturbing functions of both local tissues and the liver.^3^ Fructose intestinal metabolism implies rapid generation and accumulation of glyceraldehyde-3-phosphate and dihydroxyacetone phosphate, which are effective proglycation agents and precursors of RCS such as glyoxal and methylglyoxal, which, in turn, are precursors of more stable AGES.^10^

In the colonic tissue, the protein glycoxidation markers (PPC and APOPs) also reached the highest values, suggesting intense damage to the intestinal barrier due to the increased luminal glycoxidative stress plus the likely accretion of undesirable metabolites. Oxidative stress plays a key role in the development of IBD and cancer by the continuous exposure of the colonic cells to the intraluminal metabolic-derived free radicals.^17,62^ A comparing study about the levels of protein hydrazones in human colonic tissues with different degrees of primary colorectal tumors (colorectal adenopolyps) with their normal/surrounding tissues was carried out and highlighted that damaged tissues contained around 70 nmol hydrazones/mg protein, while healthy neighboring tissues had between 10 and 15 nmol hydrazones/mg protein.^62^ Assuming the previously mentioned equivalence factor between the sum of α-AS and γ-GS and protein hydrazones, the PPC levels of the intraluminal colonic contents from F-treated rats are close to the dangerous threshold values described by the authors in the precancerous states of CRC. The glycoxidative state in the colonic tissue was promoted by the harmful intraluminal environment. This is an important approach as it could directly link fructose consumption with colonic tissue damage.

### 4.3 Colon Microbiota, Metabolomics, and Potential Health Implications

The imbalance in gut microbiota may result in disruption of several metabolic mechanisms and immune functions, which might lead to several diseases, such as IBD, metabolic syndrome, diabetes, insulin resistance, obesity, cardiovascular diseases, and even cancer.^63^ In order to further investigate the underlying chemistry of the processes occurring in the colon of the experimental animals, comprehensive analyses of the microbiota and metabolomics of colonic digests were performed. In our study, fructose promoted alterations in the gut microbiota profile of the *Wistar* rats. Several authors have previously related the fecal microbiota shift as a consequence of an increased fructose intake.^16,64^ Nevertheless, the influence of a high-fructose diet on gut microbiota is still largely unknown. Most of the dietary fructose was metabolized in the small intestine.^60^ Moreover, liquid formulations of fructose were more rapidly absorbed and gave greater induction of hepatic lipid accumulation compared to solid counterparts.^65^ It is reasonable to hypothesize that indefinite (not analyzed in the present study) amounts of nonmetabolized fructose reached the colon of our treated animals under the experimental conditions (9 g of fructose/kg of live weight/day), and such fructose could have promoted shifts on the microbiota. Other studies in which fructose was found to reach the colon of mice registered an increase in amino acid metabolism genes in the microbiota of treated animals.^65^ In addition, it is remarkable that our results reflected that the intake of high amounts of fructose for 10 weeks increased the metabolism and/or absorption rates of protein-related glycoxylated compounds at the colonic stage, as can be inferred by the different amounts of the markers between the colonic contents and the feces. Thus, the concentration of carbonyls in the feces from the fructose group (vs control) suggested that more than 70% of the carbonylated proteins were assimilated in the colonic stage (vs 50%). Moreover, greater metabolism and/or absorption of APOPs were observed when fructose was consumed, which might support the change in the colonic protein metabolism already suggested. The higher abundance of several metabolites involved in energy metabolism, such as β-d-glucose-6-phosphate, lactic acid, or pyruvic acid in the colonic contents of the fructose rats, might support the suggested higher metabolism in the colon of F animals. In fact, pathway enrichment analysis significantly enhanced changes in glycolysis, pyruvate, and citrate cycle pathways. Some authors previously described changes in the oxidative phosphorylation pathway in plasma from fructose-consumer human volunteers,^64^ which might well be related to the intestinal events described above. However, comparisons between studies should be made with caution as the results from the aforementioned works were obtained with different experimental conditions, species, and diet formulations (solid vs liquid fructose).

The lower abundance of probiotic genera *Lactobacillus* and *Bifidobacterium* due to an enduring high-fructose intake has already been highlighted by other authors when evaluating the impact of fructose consumption on microbiota.^11,66^ Increased intestinal permeability, liver inflammation, and/or fibrosis were attributed to fructose consumption when different rats and mouse strains were exposed to tap water vs 30% fructose in drinking water for 8 weeks *ad libitum.*(57) Other authors who considered the effect of oxidized protein intake on microbiota also reported a diminished abundance of *Lactobacillus* spp.^31,67^ Furthermore, a decrease in *Bifidobacterium animalis* due to the presence of AGES in the colon was described in a review.^45^ Overall, the fructose-related decrease of probiotic bacteria could be plausibly attributed to the buildup of *in vivo* oxidized proteins in the colon as a result of the consumption of the reducing sugar. Even though other genera described as beneficial gut bacteria, such as *Adlercrautzia*(63) or *A. shashii,*(68) were slightly expressed only in the group of fructose rats, the identification of *L. grasseri* and *Bifidobacterium animalis* as species affected by fructose-liquid diet is highly relevant from the perspective of probiotic supplementation research.

The shift in microbiota observed in F rats could explain the decreased amounts of several colonic metabolites in these rats, such as acetic acid. Acetic acid production was related to the occurrence of *Lactobacillus* spp. and *Bifidobacterium* spp. in the colon by some authors.^69^ Other authors reported that some of the species from the genera *Lactobacillus* and *Streptococcus* are able to produce biogenic amines such as spermidine, described as an important compound for normal mucosa development.^69,70^ Such a metabolite was found to be significantly decreased in the colonic metabolome of *Wistar* rats exposed to dietary fructose in our study. Moreover, the capacity of some gut microorganisms to synthesize neuroactive compounds such as neurotransmitters through the catabolism of several amino acids has been described.^71^ Particularly, the authors related the gut synthesis of GABA, histamine, and serotonin with the microbial fermentation of glutamic acid, histidine, and tryptophan by genera *Lactobacillus, Bifidobacterium*, and *Streptococcus*, among others.^71^ Interestingly, in our study, the multivariant metabolomic analysis revealed that the increased amounts of tryptophan and glutamic acid in the intraluminal colonic contents of F rats were the main metabolites that explained the clustering of the samples (PLS-DA loadings, Supporting Information). Although the statistical analysis detected no changes in the abundance of histidine and serotonin between groups, our results suggest that the lower abundance of probiotic bacteria may be involved in diminishing the presence of some active compounds resulting from the degradation of amino acids, such as tryptophan, which were, in fact, increased in the colonic content of F rats. The impact of fructose on microbiota in control vs colitis-induced rats displayed that in both groups of animals, arginine and proline metabolism pathways were altered, with the expression of GABA diminished,^66^ which is in agreement with our results. However, these authors used 12.5% g of fructose in a solid-diet formula, which makes it difficult to compare the results. It is worth noting that the abundance of histamine was related with energy homeostasis and neurological disorders.^71^ Other authors described that histamine reduced the production of proinflammatory cytokines.^69^ Plausible inflammation of the intestinal mucosa could explain the significantly increased amounts of lactic acid detected in the F group (fold change: 2.24), in agreement with other findings after the measurement of the levels of lactate in feces from patients with active ulcerative colitis.^72^ Another relevant finding was the increased amount of cadaverine in the metabolomic profile of F rats (fold change: 6.27). Higher colonic levels of this polyamine, synthesized from lysine, have been linked by some authors to ulcerative colitis,^72^ but the effect of cadaverine on the colonic cells remains unknown yet.

The potential implications of protein fermentation in the gut of humans, pigs, and poultry were reviewed and some of the outcomes derived from a defective metabolism of amino acids in both the gut and the microbiota were addressed.^70^ These authors linked high expressions of sulfide-producing bacteria (i.e., *Desulfovibrio* spp., which showed an increase trend in our results) with IBD since this type of bacteria can reduce dietary sulfide and sulfate and sulfated polysaccharides from mucins, decreasing mucus barrier integrity in IBD.^70^ The decreased amounts of cysteine (fold change: −3.26; *p*-value <0.001) observed in the intracolonic metabolome of treated rats might be related to the growth of the sulfate-reducing bacteria. Fructose has been proven to be associated with impaired mucus production by enterocytes.^66^ The mechanisms remain unclear, but the decreased protein digestibility promoted by fructose intake could be responsible for the increased expression of genera *Desulfovibrio* at the colon stage, which, in turn, might be involved in the impairment of the mucosa along with the other changes described. Another study about the impact of protein oxidation on microbiota revealed an increased presence of *Desulfovibrio* spp. after the intake of oxidized meat proteins.^67^

Uncultured *Lachnospiraceae* spp. and unclassified *Marvynbryantia* bacteria were increased in the microbiota of F rats in our experiment. Accordingly, an increased abundance of genera of the *Lachnospiraceae* family in Sprague-Dawley (SD) rats exposed to different doses of fructose during 20 weeks was assessed.^73^ The fructose dose that promoted the increase of *Lachnospira* spp. and *Marvynbryantia* spp. in that study is similar to that used in the present assay (10.5 g/kg/day). The intake of fructose also increased unclassified genera of the *Lachnospiraceae* family in a comparative study,^66^ where the authors attributed the changes in microbiota to fructose intake rather than induced colitis, which is in agreement with our results. Interestingly, other authors that evaluated the effect of the intake of high amount of cured meat-derived proteins on the microbiota described an increased *Lachnospiraceae* spp.^31^ The *Lachnospiraceae* family has been reported to be butyrate-producing bacteria that may protect the intestinal epithelium from inflammation.^70,74^ Moreover, the *Marvynbryantia* and *Christensenelleceae R-7 groups*, also increased in the microbiome of our F rats, were associated in humans with a lower insulin index and lower BMI in human research.^75^ The *Christensenelleceae R-7 group* was decreased in populations that consumed a high-fructose corn syrup-based diet.^76^

The microbiome of the F rats showed an increase in uncultured *Ruminococcaceae* spp. Several studies that made associations between increased *Ruminococcacea* with fructose-rich diets and liver disease (i.e., NAFLD) were reviewed.^77^ On the other side, other authors described increased colonic *Ruminoccocaceae* related to the intake of oxidized proteins from cured meat consumption.^31^ Anyway, members of the *Ruminococcaceae* family can expand as a consequence of a high availability of proteins.^78^

Likewise, it would be the first full assessment of the *in vivo* glycoxidative stress promoted by fructose during gastrointestinal digestion and its relevant impact on the intraluminal protein and amino acid metabolism, which may be related to immunity and proinflammatory functions.^66^ The intake of 9 g of fructose/kg of live weight/day for 10 weeks strongly affects the fate of dietary proteins during digestion in *Wistar* rats. The glycoxidative environment promoted by the reducing sugar at the first stages of the GIT condition the whole intraluminal protein digestion. Glycoxidative markers are increased along the digestion, and the surrounding tissues are affected. At the colon stage, fructose and its promoted protein-degradation products (i.e., carbonyls and AGES) increase the glycoxidative environment and have an impact on the microbiota and the metabolomic fingerprint, boosting an amino acidic dysbiosis that could be the basis of the microbiota shift and the related mucosal inflammation and metabolic disorders. Thus, fructose intake decreases the expression of probiotic bacteria as well as the abundance of biogenic amines with neurotransmitter properties while enhancing the expression of sulfate-reducing bacteria and harmful metabolites.

## Supporting Information Available

Energy (kJ/day) provided by the feed and the supplemented water to the experimental animals during the assay (Table S1), weight evolution of the animals during the experiment (Table S2), list of species of microorganisms selected to statistical analysis based on the literature (Table S3), and described metabolite characterization (Table S4) (PDF)
Relative abundance of selected species of microorganisms (L6), volcano results with the changes in the abundance of colonic metabolites and statistical significance (Volcano), and PLS-DA loadings list from the colonic metabolome with the component coordinates (PLS-DA loadings) (XLSX)

## Abbreviations

GIT: gastrointestinal tract
WHO: World Health Organization
HFCS: high-fructose corn syrup
MetS: metabolic syndrome
NAFLD: nonalcoholic fatty liver disease
T2D: type 2 diabetes mellitus
RCS: reactive carbonyls species
AGES: advanced glycation end products
IBD: inflammatory bowel disease
CRC: colorectal cancer
C: control group
F: fructose group
α-AS: α-aminoadipic semialdehyde
γ-GS: γ-glutamic semialdehyde
PPC: primary protein carbonyls
APOPs: advanced protein oxidation products
FU: fluorescent units
TBARS: thiobarbituric reactive substances
MDA: malondialdehyde
BHT: butylated hydroxytoluene
TBA: thiobarbituric acid
TEP: 1,1,3,3-tetraethoxypropane
AOAC: Association of Official Agricultural Chemists
TN: total nitrogen
WSN: water-soluble nitrogen
NPN: nonprotein nitrogen
TCA: trichloroacetic acid
TPN: total protein nitrogen
TDN: total dietary nitrogen
Ep: endogenous nitrogen
TDP: total dietary protein
DP: digested proteins
FP: fermented proteins
PLS-DA: partial least-squares discriminant analysis
VIP: variable importance in projection
FC: fold change
TPD: true protein digestibility
OTUs: operational taxonomic units
ROS: reactive oxygen species
S: stage
D: diet
