# Supplementary material for: Impact of Sustained Fructose Consumption on Gastrointestinal Function and Health in Wistar Rats: Glycoxidative Stress, Impaired Protein Digestion, and Shifted Fecal Microbiota
Source: J Agric Food Chem. 2023 Oct 20;71(43):16270–85. doi: 10.1021/acs.jafc.3c04515 (PMC10623553; doi:10.1021/acs.jafc.3c04515)
Supplement: Supplementary file 1 — jf3c04515_si_001.pdf [file jf3c04515_si_001.pdf]

Supporting Information

Impact of Sustained Fructose Consumption on Gastrointestinal Function and Health in *Wistar* rats: Glycoxidative Stress, Impaired Protein Digestion and Shifted Faecal Microbiota

Guadalupe Sánchez-Terrón[1], Remigio Martínez[1,2,3], Jorge Ruiz[1], Carolina Luna[4], & Mario Estévez[1*]

[1] TECAL Research Group, Meat and meat products research institute (IPROCAR), Universidad de Extremadura (UEX, ROR-ID 0174shg90), Cáceres, 10003, Spain

[2] Animal Health Department. Universidad of Extremadura (UEX, ROR-ID 0174shg90), Cáceres, 10003, Spain.

[3] Animal Health Department, GISAZ Research Group, ENZOEM Competitive Research Unit, Universidad of Córdoba (UCO, ROR-ID 05yc77b46), Córdoba, 14014, Spain.

[4] Emergency unit, Servicio Extremeño de Salud, SES, Junta de Extremadura (ROR-ID 0174shg90), Cáceres, 10003, Spain

*Corresponding author: Mario Estévez, Email: mariovet@unex.es

TABLE S1. Energy (kJ/day) provided by the feed and the supplemented water expressed as median [interquartile range] in the experimental consumer groups.

	Feed energy[a]	Fructose-drinking water energy[a]	Total energy[a]
Fructose	87.41 [141.71-56.80]	39.41 [57.77-28.51]	131.90 [188.48-95.51]
Control	99.41 [151.78-72.19]	0.00 [0.00-0.00]	99.41 [151.78-72.19]
p value[b]	ns	***	ns

[a] Energy supplied by the feed or/and the fructose-drinking water expressed in kJ/day.

[b] Significance level in U-Mann Whitney test with the effect of fructose supplementation. *: $p<0.05$; **: $p<0.01$; ***: $p<0.001$; ns: no significant.

TABLE S2. Evolution of the weights of the fructose and control rats during the study.

Week	Group	Weight[a]	*p* value[b]
0	Fructose	177.97±22.18	ns
	Control	175.48±18.08	
1	Fructose	225.00±23.88	ns
	Control	229.25±19.98	
2	Fructose	269.69±26.88	ns
	Control	271.18±27.50	
3	Fructose	303.79±29.56	ns
	Control	296.00±31.24	
4	Fructose	332.61±31.50	ns
	Control	313.28±33.36	
5	Fructose	352.76±33.62	ns
	Control	332.23±32.09	
6	Fructose	373.16±36.44	ns
	Control	355.08±35.25	
7	Fructose	404.16±41.90	ns
	Control	379.85±32.67	
8	Fructose	412.10±45.01	ns
	Control	390.12±29.96	
9	Fructose	433.17±46.46	ns
	Control	404.52±29.88	
10	Fructose	441.56±45.38	ns
	Control	410.35±24.61	

[a] Means±SD of the animal weights during the experiment expressed as g.

[b] Significance level in Student-T test with the effect of the diet (fructose and control). *: $p<0.05$; **: $p<0.01$; ***: $p<0.001$; ns: no significant.

TABLE S3. List of species selected for the statistical analysis to highlighted differences among the relative abundance of *bacterium* from genera affected by the fructose treatment.

Genera	Detected species	Criteria	References
Akkermansia	Uncultured bacterium	Fructose-affected proposed microorganism	1
Lactobacillus	Unclassified bacterium<br>*Lactobacillus grasseri*<br>*Lactobacillus reuteri*<br>*Lactobacillus intestinalis*	Fructose-affected proposed microorganism<br>Oxidized protein-affected proposed microorganism	1,2
Escherichia-Shigella	Unclassified bacterium	Proteolytic bacteria	3
Bacteroides	Unclassified bacterium<br>*Bacteroides acidifaciens*<br>*Bacteroides vulgatus*<br>*Bacteroides uniformis*<br>*Bacteroides eggerthii*<br>*Bacteroides massiliensis*<br>*Bacteroides intestinalis*	Fructose-affected proposed microorganism<br>Proteolytic bacteria	1,3
Ruminococcus	Unidentified bacterium<br>Unclassified bacterium<br>*Ruminococcus flavefaciens*<br>*Ruminococcus callidus*	Fructose-affected proposed microorganism	4
Alistipes	Uncultured bacterium<br>Unclassified *Alistipes*<br>*Alistipes finegoldii*<br>*Alistipes shashii*	Proteolytic bacteria	3
Parasuterella	Unclassified bacterium	Fructose-affected proposed microorganism	4
Romboutsia	Unclassified bacterium		
Muribaculum	Unclassified bacterium<br>Uncultured bacterium	Fructose-affected proposed microorganism	1
Clostridium sensu stricto 1	Unclassified bacterium	Proteolytic bacteria<br>Oxidized protein-affected proposed microorganism	2,3
Erysipelatoclostridium	Unclassified bacterium	Fructose-affected proposed microorganism Proteolytic bacteria	3,4
Barnesiella	Unclassified bacterium	Fructose-affected proposed microorganism	4
Parabacteroides	*Parabacteroides sp.*<br>*Parabacteroides merdae*<br>*Parabacteroides distasonis*	Fructose-affected proposed microorganism Proteolytic bacteria	3,4
Blautia	Lachnospiraceae bacterium<br>Unclassified bacterium		
Roseburia	Uncultured bacterium<br>Unclassified bacterium		
Marvinbryantia	Uncultured bacterium<br>Unclassified bacterium	Fructose-induced microbiota shift	1
Lachnoclostridium	Unclassified bacterium	Proteolytic bacteria	3
Streptococcus	*Streptococcus sp.*		
Bifidobacterium	*Bifidobacterium animalis*	AGEs[1]-affected proposed microorganism	5

[1]AGEs: Advanced glycation end-products.

TABLE S4. Metabolites characterization.

Name	Formula	Structure	Cal. MW	m/z	MS2	Reference ion	SMILES	InChi
Phenylalanine	C9 H11 N O2	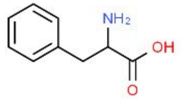	165.078	166.086	DDA for preferred ion	[M+H]+1	c1ccc(cc1)CC(C(=O)O)N	InChI=1S/C9H11NO2/c10-8(9(11)12)6-7-4-2-1-3-5-7/h1-5,8H,6,10H2,(H,11,12)
alpha-Cyclopentylmandelic acid	C13 H16 O3	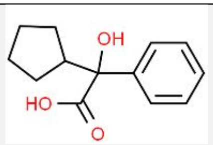	220.109	221.116	No MS2	[M+H]+1	c1ccc(cc1)C(C2CCCC2)(C(=O)O)O	InChI=1S/C13H16O3/c14-12(15)13(16,11-8-4-5-9-11)10-6-2-1-3-7-10/h1-3,6-7,11,16H,4-5,8-9H2,(H,14,15)
Histamine	C5 H9 N3	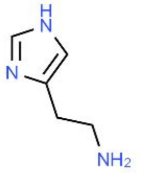	111.079	112.086	No MS2	[M+H]+1	c1c(nc[nH]1)CCN	InChI=1S/C5H9N3/c6-2-1-5-3-7-4-8-5/h3-4H,1-2,6H2,(H,7,8)
Inspra	C24 H30 O6	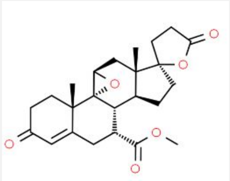	414.203	415.211	No MS2	[M+H]+1	C[C@]12CCC(=O)C=C1C[C@H]([C@@H]3[C@]24[C@H](O4)C[C@]5([C@H]3CC[C@@]56CCC(=O)O6)C)C(=O)OC	InChI=1S/C24H30O6/c1-21-7-4-14(25)10-13(21)11-15(20(27)28-3)19-16-5-8-23(9-6-18(26)30-23)22(16,2)12-17-24(19,21)29-17/h10,15-17,19H,4-9,11-12H2,1-3H3/t15-,16+,17-,19+,21+,22+,23-,24-/m1/s1
Creatine	C4 H9 N3 O2	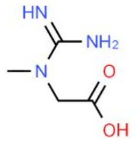	131.069	132.076	DDA for preferred ion	[M+H]+1	CN(CC(=O)O)C(=N)N	InChI=1S/C4H9N3O2/c1-7(4(5)6)2-

								3(8)9/h2H2,1H3,(H3,5,6)(H,8,9)
Phosphoric acid	H3 O4 P	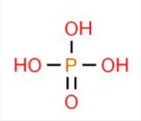	97.976	96.969	DDA for preferred ion	[M-H]-1	OP(=O)(O)O	InChI=1S/H3O4P/c1-5(2,3)4/h(H3,1,2,3,4)
(9Z)-9-Octadecenamide	C18 H35 N O	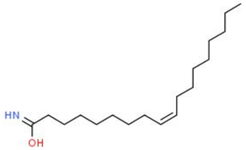	281.271	282.278	No MS2	[M+H]+1	CCCCCCCC/C=C\CCCCCCCC(=N)O	InChI=1S/C18H35NO/c1-2-3-4-5-6-7-8-9-10-11-12-13-14-15-16-17-18(19)20/h9-10H,2-8,11-17H2,1H3,(H2,19,20)/b10-9-
2-(Trimethylammonio) ethanolate	C5 H13 N O	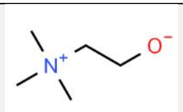	103.099	104.106	DDA for preferred ion	[M+H]+1	C[N+](C)(C)CC[O-]	InChI=1S/C5H13NO/c1-6(2,3)4-5-7/h4-5H2,1-3H3
beta-Alanine	C3 H7 N O2	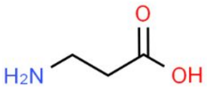	89.047	88.040	DDA for preferred ion	[M-H]-1	C(CN)C(=O)O	InChI=1S/C3H7NO2/c4-2-1-3(5)6/h1-2,4H2,(H,5,6)
(1,1-Dioxido-1,2-thiazinan-3-yl) methanol	C5 H11 N O3 S	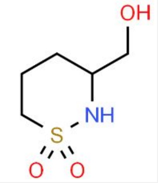	165.045	166.053	DDA for preferred ion	[M+H]+1	C1CC(NS(=O)(=O)C1)CO	InChI=1S/C5H11NO3S/c7-4-5-2-1-3-10(8,9)6-5/h5-7H,1-4H2
(1R,2R,3R,4S,5R)-4-Amino-5-sulfanyl-1,2,3-cyclopentanetriol	C5 H11 N 03 S	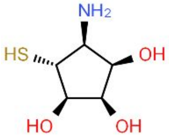	165.045	166.053	DDA for preferred ion	[M+H]+1	[C@@H]1([C@H]([C@H]([C@H]([C@@H]1S)O)O)O)N	InChI=1S/C5H11NO3S/c6-1-2(7)3(8)4(9)5(1)10/h1-5,7-10H,6H2/t1-,2+,3+,4+,5+/m0/s1

2-Methylamino-butyric acid	C5 H11 N 02	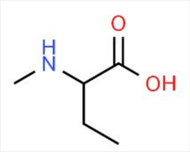	117.078	118.086	DDA for preferred ion	[M+H]+1	O=C(O)C(NC)CC	InChI=1S/C5H11NO2/c1-3-4(6-2)5(7)8/h4,6H,3H2,1-2H3,(H,7,8)
Glutamic acid	C5 H9 N 04	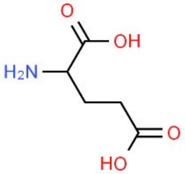	147.053	148.060	DDA for preferred ion	[M+H]+1	C(CC(=O)O)C(C(=O)O)N	InChI=1S/C5H9NO4/c6-3(5(9)10)1-2-4(7)8/h3H,1-2,6H2,(H,7,8)(H,9,10)
Proline	C5 H9 N 02	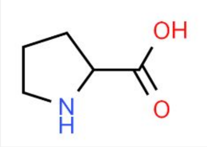	115.063	116.070	DDA for preferred ion	[M+H]+1	C1CC(NC1)C(=O)O	InChI=1S/C5H9NO2/c7-5(8)4-2-1-3-6-4/h4,6H,1-3H2,(H,7,8)
Cyclandelate	C17 H24 O3	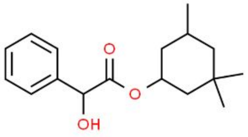	276.172	277.179	No MS2	[M+H]+1	CC1CC(CC(C1)(C)C)OC(=O)C(c2ccccc2)O	InChI=1S/C17H24O3/c1-12-9-14(11-17(2,3)10-12)20-16(19)15(18)13-7-5-4-6-8-13/h4-8,12,14-15,18H,9-11H2,1-3H3
Sulfuric acid	H2 04 S	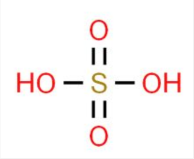	97.967	96.960	DDA for preferred ion	[M-H]-1	OS(=O)(=O)O	InChI=1S/H2O4S/c1-5(2,3)4/h(H2,1,2,3,4)
Linoelaidic Acid	C18 H32 02	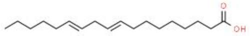	280.239	281.246	No MS2	[M+H]+1	CCCCC/C=C/C/C=C/CCCCCCCC(=O)O	InChI=1S/C18H32O2/c1-2-3-4-5-6-7-8-9-10-11-12-13-14-15-16-17-18(19)20/h6-7,9-10H,2-

								5,8,11-17H2,1H3,(H,19,20)/b7-6+,10-9+
Nonivamide	C17 H27 N O3	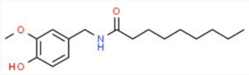	293.198	294.205	DDA for preferred ion	[M+H]+1	CCCCCCCCC(=O)NCc1ccc(c(c1)OC)O	InChI=1S/C17H27NO3/c1-3-4-5-6-7-8-9-17(20)18-13-14-10-11-15(19)16(12-14)21-2/h10-12,19H,3-9,13H2,1-2H3,(H,18,20)
L-Palmitin	C19 H38 O4	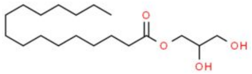	330.276	331.283	No MS2	[M+H]+1	CCCCCCCCCCCCCCCC(=O)OCC(CO)O	InChI=1S/C19H38O4/c1-2-3-4-5-6-7-8-9-10-11-12-13-14-15-19(22)23-17-18(21)16-20/h18,20-21H,2-17H2,1H3
Linoleamide	C18 H33 N O	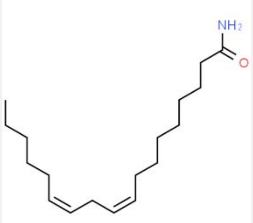	279.255	280.262	No MS2	[M+H]+1	CCCCC/C=C\C/C=C\CCCCCCCC(=O)N	InChI=1S/C18H33NO/c1-2-3-4-5-6-7-8-9-10-11-12-13-14-15-16-17-18(19)20/h6-7,9-10H,2-5,8,11-17H2,1H3,(H2,19,20)/b7-6-,10-9-
1-Dodecyl-2-pyrrolidinone	C16 H31 N O	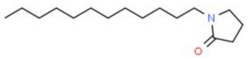	253.240	254.247	No MS2	[M+H]+1	CCCCCCCCCCCCN1CCCC1=O	InChI=1S/C16H31NO/c1-2-3-4-5-6-7-8-9-10-11-14-17-15-12-13-16(17)18/h2-15H2,1H3
Indane	C9 H10	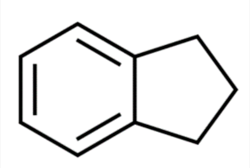	118.078	119.085	No MS2	[M+H]+1	c1ccc2c(c1)CCC2	InChI=1S/C9H10/c1-2-5-9-7-3-6-8(9)4-1/h1-2,4-5H,3,6-7H2

1-(6-Methoxy-2-naphthyl) ethanol	C13 H14 O2	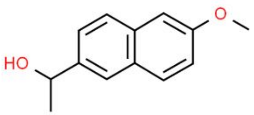	202.099	203.106	No MS2	[M+H]+1	C[C@H](c1ccc2cc(ccc2c1)OC)O	InChI=1S/C13H14O2/c1-9(14)10-3-4-12-8-13(15-2)6-5-11(12)7-10/h3-9,14H,1-2H3/t9-/m1/s1
Oleic acid	C18 H34 O2	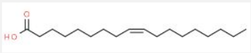	282.255	283.262	DDA for preferred ion	[M+H]+1	CCCCCCCC/C=C\CCCCCCCC(=O)O	InChI=1S/C18H34O2/c1-2-3-4-5-6-7-8-9-10-11-12-13-14-15-16-17-18(19)20/h9-10H,2-8,11-17H2,1H3,(H,19,20)/b10-9-
1-Octen-3-yne	C8 H12	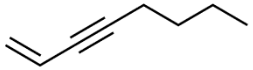	108.093	109.101	DDA for preferred ion	[M+H]+1	CCCCC#CC=C	InChI=1S/C8H12/c1-3-5-7-8-6-4-2/h3H,1,4,6,8H2,2H3
[1-Amino-1-(2-thienyl) ethyl] phosphinic acid	C6 H10 N O2 P S	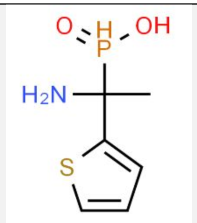	191.016	192.024	DDA for preferred ion	[M+H]+1	CC(c1cccs1)(N)P(=O)O	InChI=1S/C6H10NO2PS/c1-6(7,10(8)9)5-3-2-4-11-5/h2-4,10H,7H2,1H3,(H,8,9)
3-Oxostearic acid	C18 H34 03	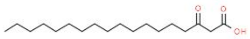	298.250	299.257	DDA for preferred ion	[M+H]+1	CCCCCCCCCCCCCCCC(=O)CC(=O)O	InChI=1S/C18H34O3/c1-2-3-4-5-6-7-8-9-10-11-12-13-14-15-17(19)16-18(20)21/h2-16H2,1H3,(H,20,21)

4-Aminopyridine	C5 H6 N2	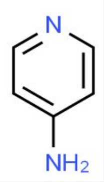	94.053	95.060	No MS2	[M+H]+1	c1c[nH]ccc1=N	InChI=1S/C5H6N2/c6-5-1-3-7-4-2-5/h1-4H,(H2,6,7)
1-Benzosuberone	C11 H12 O	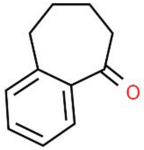	160.088	161.095	No MS2	[M+H]+1	c1ccc2c(c1)CCCCC2=O	InChI=1S/C11H12O/c12-11-8-4-2-6-9-5-1-3-7-10(9)11/h1,3,5,7H,2,4,6,8H2
2,2'-(Dodecylimino) diethanol	C16 H35 N O2	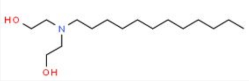	273.266	274.273	No MS2	[M+H]+1	CCCCCCCCCCCCN(CCO)CCO	InChI=1S/C16H35NO2/c1-2-3-4-5-6-7-8-9-10-11-12-17(13-15-18)14-16-19/h18-19H,2-16H2,1H3
beta-D-glucose 6-phosphate (6-O-Phosphono-beta-D-glucopyranose)	C6 H13 09 P	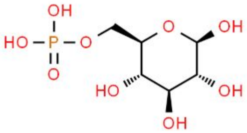	260.029	259.022	DDA for preferred ion	[M-H]-1	C([C@@H]1[C@H]([C@@H]([C@H]([C@H](O1)O)O)O)O)OP(=O)(O)O	InChI=1S/C6H13O9P/c7-3-2(1-14-16(11,12)13)15-6(10)5(9)4(3)8/h2-10H,1H2,(H2,11,12,13)/t2-,3-,4+,5-,6+/m1/s1
2-Aminobutanoic acid	C4 H9 N O2	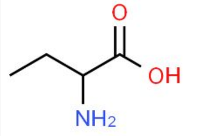	103.063	104.070	DDA for preferred ion	[M+H]+1	CCC(C(=O)O)N	InChI=1S/C4H9NO2/c1-2-3(5)4(6)7/h3H,2,5H2,1H3,(H,6,7)
Cadaverine	C5 H14 N2	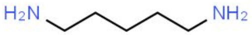	102.115	103.122	DDA for preferred ion	[M+H]+1	C(CCN)CCN	InChI=1S/C5H14N2/c6-4-2-1-3-5-7/h1-7H2
Prolylleucylglycine	C13 H23 N3 O4	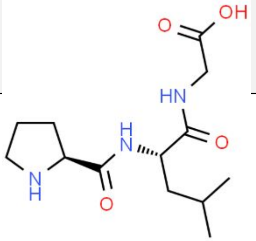	285.168	286.175	No MS2	[M+H]+1	CC(C)C[C@@H](C(=O)NCC(	InChI=1S/C13H23N3O4/c1-8(2)6-10(12(19)15-7-

							=O)O)NC(=O)[C@@H]1CCCN1	11(17)18)16-13(20)9-4-3-5-14-9/h8-10,14H,3-7H2,1-2H3,(H,15,19)(H,16,20)(H,17,18)/t9-,10-/m0/s1
Serylglycine	C5 H10 N2 O4	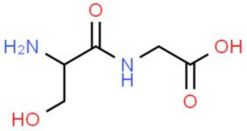	162.063	163.071	DDA for preferred ion	[M+H]+1	C(C(C(=O)NCC(=O)O)N)O	InChI=1S/C5H10N2O4/c6-3(2-8)5(11)7-1-4(9)10/h3,8H,1-2,6H2,(H,7,11)(H,9,10)
Pipecolic acid	C6 H11 N O2	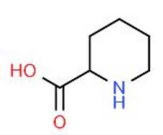	129.078	130.086	DDA for preferred ion	[M+H] +1	C1CCNC(C1)C(=O)O	InChI=1S/C6H11NO2/c8-6(9)5-3-1-2-4-7-5/h5,7H,1-4H2,(H,8,9)
Pyruvic acid	C3 H4 O3	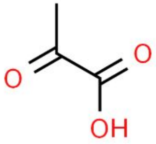	88.016	87.008	DDA for preferred ion	[M-H]-1	CC(=O)C(=O)O	InChI=1S/C3H4O3/c1-2(4)3(5)6/h1H3,(H,5,6)
Lactic acid	C3 H6 O3	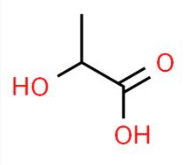	90.031	89.024	DDA for preferred ion	[M-H]-1	CC(C(=O)O)O	InChI=1S/C3H6O3/c1-2(4)3(5)6/h2,4H,1H3,(H,5,6)

Tryptophan	C11 H12 N2 O2	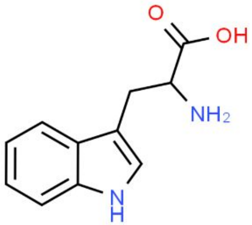	204.089	205.096	No MS2	[M+H]+1	c1ccc2c(c1)c(c[nH]2)CC(C(=O)O)N	InChI=1S/C11H12N2O2/c12-9(11(14)15)5-7-6-13-10-4-2-1-3-8(7)10/h1-4,6,9,13H,5,12H2,(H,14,15)
2-Oxobutyric acid	C4 H6 O3	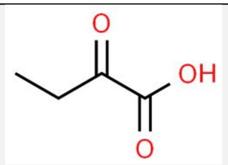	102.031	101.02	DDA for preferred ion	[M-H]-1	CCC(=O)C(=O)O	InChI=1S/C4H6O3/c1-2-3(5)4(6)7/h2H2,1H3,(H,6,7)
Spermidine	C7 H19 N3	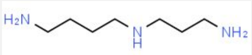	145.157	146.165	No MS2	[M+H]+1	C(CCNCCCN)CN	InChI=1S/C7H19N3/c8-4-1-2-6-10-7-3-5-9/h10H,1-9H2
Hypotaurine	C2 H7 N O2 S	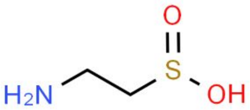	109.019	110.026	DDA for preferred ion	[M+H]+1	C(CS(=O)O)N	InChI=1S/C2H7NO2S/c3-1-2-6(4)5/h1-3H2,(H,4,5)
Acetic acid	C2 H4 O2	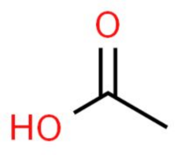	60.020	59.013	DDA for preferred ion	[M-H]-1	CC(=O)O	InChI=1S/C2H4O2/c1-2(3)4/h1H3,(H,3,4)

2,6-Diaminopimelic acid	C7 H14 N2 O4	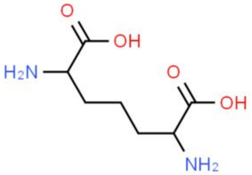	190.095	189.088	DDA for preferred ion	[M-H]-1	C(CC(C(=O)O)N)CC(C(=O)O)N	InChI=1S/C7H14N2O4/c8-4(6(10)11)2-1-3-5(9)7(12)13/h4-5H,1-3,8-9H2,(H,10,11)(H,12,13)
Maleic acid	C4 H4 O4	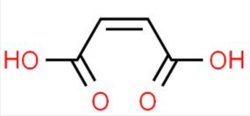	116.010	115.003	DDA for preferred ion	[M-H]-1	C(=C\C(=O)O)\C(=O)O	InChI=1S/C4H4O4/c5-3(6)1-2-4(7)8/h1-2H,(H,5,6)(H,7,8)/b2-1-
Glyceraldehyde	C3 H6 O3	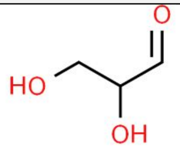	90.031	89.024	DDA for preferred ion	[M-H]-1	C(C(C=O)O)O	InChI=1S/C3H6O3/c4-1-3(6)2-5/h1,3,5-6H,2H2
gamma-Aminobutyric acid	C4 H9 N O2	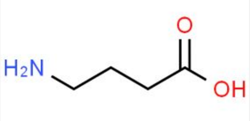	103.063	104.070	No MS2	[M+H]+1	C(CC(=O)O)CN	InChI=1S/C4H9NO2/c5-3-1-2-4(6)7/h1-3,5H2,(H,6,7)
Glycerol 3-phosphate	C3 H9 O6 P	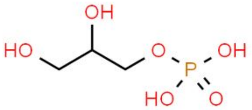	172.013	195.002	DDA for preferred ion	[M+Na]+1	C(C(COP(=O)(O)O)O)O	InChI=1S/C3H9O6P/c4-1-3(5)2-9-10(6,7)8/h3-5H,1-2H2,(H2,6,7,8)
Dihydroxyphenyl alanine	C9 H11 N O4	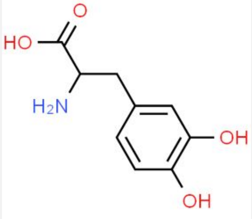	179.068	198.075	DDA for preferred ion	[M+H]+1	c1cc(c(cc1CC(C(=O)O)N)O)O	InChI=1S/C9H11NO4/c10-6(9(13)14)3-5-1-2-7(11)8(12)4-5/h1-2,4,6,11-12H,3,10H2,(H,13,14)

References

(1) Kawano, Y.; Edwards, M.; Huang, Y.; Bilate, A. M.; Araujo, L. P.; Tanoue, T.; Atarashi, K.; Ladinsky, M. S.; Reiner, S. L.; Wang, H. H.; Mucida, D.; Honda, K.; Ivanov, I. I. Microbiota Imbalance Induced by Dietary Sugar Disrupts Immune-Mediated Protection from Metabolic Syndrome. *Cell* **2022**, *185* (19), 3501-3519.e20. https://doi.org/10.1016/j.cell.2022.08.005.

(2) Yin, Y.; Cai, J.; Zhou, L.; Xing, L.; Zhang, W. Dietary Oxidized Beef Protein Alters Gut Microbiota and Induces Colonic Inflammatory Damage in C57BL/6 Mice. *Front. Nutr.* **2022**, *9* (3). https://doi.org/10.3389/fnut.2022.980204.

(3) Oliphant, K.; Allen-Vercoe, E. Macronutrient Metabolism by the Human Gut Microbiome: Major Fermentation by-Products and Their Impact on Host Health. *Microbiome* **2019**, *7* (1), 1–15. https://doi.org/10.1186/s40168-019-0704-8.

(4) Beisner, J.; Gonzalez-granda, A.; Basrai, M.; Damms-machado, A. Fructose-Induced Intestinal Microbiota Shift. *Nutrients* **2020**, *12*, 1–21.

(5) Snelson, M.; Coughlan, M. T. Dietary Advanced Glycation End Products: Digestion, Metabolism and Modulation of Gut Microbial Ecology. *Nutrients* **2019**, *11* (2). https://doi.org/10.3390/nu11020215.
